# Analyzing Spectral Similarities for Structural Identification Using a New Benchmark Database

**DOI:** 10.1021/acs.jpca.5c06253

**Published:** 2026-01-12

**Authors:** Rami Rahimi, Noga Saban, Ilana Bar

**Affiliations:** Department of Physics, 26732 Ben-Gurion University of the Negev, Beer-Sheva 8410501, Israel

## Abstract

Vibrational spectra, characterized by structurally sensitive features, offer critical insights into molecular structures, bonding, and dynamics. Yet, interpreting measured spectra and identifying corresponding structures require theoretical equivalents and quantitative analysis. Here, we introduce a new experimental database that includes broad-range ionization-detected stimulated Raman scattering signatures besides harmonic Raman frequencies calculated with widely used density functional methods/basis sets. By comparing experimental fundamental bands and computed data, we derive single global and multiple range- and mode-dependent scaling factors and analyze the resulting error distributions, showing that mode-dependent scaling provides the greatest accuracy. Additionally, we explore various methods for evaluating similarities between measured fundamental spectra of different compounds and calculated data sets of conformers. Our findings indicate that Euclidean and Manhattan distance metrics for frequencies and intensities uncover subtle structural variations, yielding spectral similarity rankings that facilitate structural identifications. This new database and methodology address key challenges in spectral assignment, and we anticipate that they will serve as benchmarks for future predictive models and foster the development of advanced strategies.

## Graphical abstract

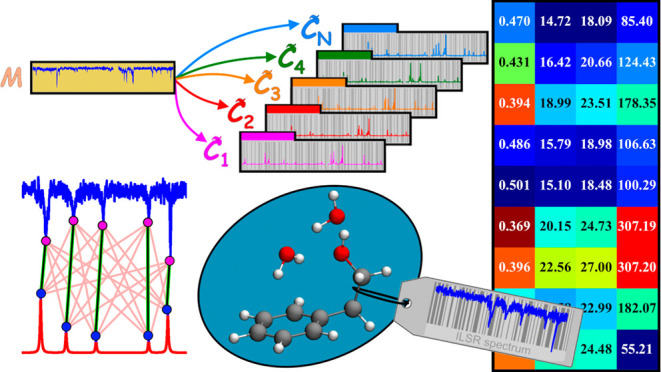

## Introduction

Vibrational spectroscopies, including spontaneous Raman and infrared (IR) spectroscopies and their various enhanced and nonlinear derivatives, provide signatures that reflect the oscillatory motion of compounds. These powerful techniques require matching unknown experimental spectra with corresponding spectral signatures found in reference-measured spectral databases, playing a significant role in characterizing compounds in different scientific disciplines.^1,2^ To manage the complexities of rich spectra, spectral matching is often performed with machine-learning algorithms and chemometric models.^3–6^

Moreover, there is a growing interest in the observable features and patterns revealed by these methods, which require quantum chemical methods for molecular and structural characterization.^7,8^ This support frequently comes from IR and Raman spectra calculations within the harmonic approximation, balancing computational cost, and tolerable accuracy. These calculations involve evaluating the second derivative of the energy with respect to nuclear coordinates at the optimized structure and determining intensities from changes in the dipole moment or polarizability along the respective normal-mode vectors. Typically, they are performed using density functional theory (DFT)^9–11^ or more computationally demanding quantum *ab initio* methods,^12,13^ depending on the chosen approximations.

To correct the systematic overestimation of harmonic frequencies, single or region-dependent empirical scaling factors are commonly applied,^14–20^ improving agreement with experimental data. Alternatively theoretical models such as vibrational self-consistent field^21–24^ and second-order vibrational theory (VPT2),^25,26^ enable the calculation of anharmonic frequencies, providing a more comprehensive description of the anharmonic nature of vibrations near a well-defined equilibrium structure. In addition, IR or Raman spectra can be calculated by *ab initio* molecular dynamics,^23,24,27–30^ which is particularly suited for capturing molecular flexibility, temperature effects, and their influence on the overall spectral profile. By comparing results from different computational methods with experimental vibrational spectra, specifically subsets of spectral peaks corresponding to fundamental transitions, one can assess their agreement. This comparison ultimately identifies the calculated spectrum that best matches the experimental data, providing detailed structural insights.

DFT is a standard and widely used method for vibrational spectra calculations and is also applied in specialized areas such as double-resonance hole-burn techniques.^11^ These methods include infrared-ion dip spectroscopy (IR-IDS)^31–33^ and the more complex ionization-detected stimulated Raman spectroscopy,^34,35^ which we pioneered for structural analysis. These methods use a single (IR) or two (pump, ω_p_, and tunable Stokes, ω_S_) laser beams to deplete the vibrational ground state population resonantly and probe it by a delayed ultraviolet (UV) laser beam, enabling resonance-enhanced two-photon ionization (R2PI). The stimulated Raman scattering (SRS) can induce depletion or gain of the background ionization signal, Figure S1 of the Supporting Information.^34,35^ These approaches facilitate species- and structure-specific jet-cooled vibrational signatures at low concentrations, offering mass-selectivity, high sensitivity, and spectral resolution higher than those of condensed phases.

Nevertheless, IR-IDS measurements for various gas-phase compounds are mainly limited to hydride stretching spectral ranges.^31–33^ Yet, efforts to capture low-frequency vibrational signatures using IR-IDS, by radiation from the free electron laser for IR experiments (FELIX),^36,37^ or ionization-loss stimulated Raman spectroscopy (ILSRS), Figure S1a of the Supporting Information, by table-top visible lasers^38–41^ have been proven effective. Consequently, the resulting vibrational bands are well-suited for comparison with theoretical predictions derived from harmonic approaches with empirical scaling, emphasizing visual agreement between the measured fundamental bands in the spectra and the calculated vibrational spectra. A notable example of this challenge is the spectral identification and assignment of conformers of neurotransmitters, analogs, and their hydrates.^34,35,38–46^ These species consist of a rigid skeleton with a flexible ethylamino (ethyl alcohol) side chain, which can undergo internal rotations about the C–N­(O) and C–C single bonds or attach water differently, leading to different conformers.

As highlighted by Mata and Suhm,^47^ benchmark experiments, specifically those conducted under gas-phase isolation and at low temperatures, are essential for validating theoretical predictions. A recently introduced resource, VIBFREQ1295, offers a valuable data set for benchmarking vibrational frequency calculations and training machine-learning models.^20^ It includes 1295 experimental fundamental frequencies compiled from a thorough review of recent literature alongside CCSD­(T)­(F12*)/cc-pVDZ-F12 *ab initio* harmonic frequencies for 141 small-to-medium-sized gas-phase molecules. While this database is highly useful, we propose an additional data set based on high quality, internally consistent data we have collected over the years.^34,35,38–46^ Although not exhaustive across all molecular types or environments, our data set includes jet-cooled ionization-loss and -gain SRS features of the aforementioned conformers (400–3755 cm^–1^)^34,35,38–46^ along with harmonic vibrational frequencies calculated using various density functionals. Based on visual similarities between the signatures obtained by comparing patterns and wavenumbers of fundamental features to those of predicted ones, we derive single global or multiple frequency region- and mode-dependent scaling factors and analyze the resulting error distributions.

Since different computational methods and basis sets introduce systematic errors in harmonic vibrational frequencies, tailored scaling factors are needed to correct these biases. Moreover, as higher-quality experimental data become available, they enable the extraction of updated global and multiple scaling factors, which in turn help extend the predictive accuracy, particularly for flexible conformers and complex molecular systems.

In the next phase, we evaluate the similarities between the measured fundamental bands in the spectra of specific conformers and the predicted scaled harmonic Raman spectra for possible conformers. To achieve this, we use multiple approaches in machine learning, including cross-correlation, Euclidean and Manhattan distances, and optimal transport^48^ and the Kuhn-Munkres algorithm (Hungarian method).^49,50^ The cross-correlation technique identifies the patterns and relationships within data sets by quantifying the degree of similarity between the spectral features. To measure discrete differences between spectra, Euclidean and Manhattan distances as well as optimal transport metrics are employed. The Kuhn-Munkres algorithm is then applied to construct minimal cost matrixes, enabling the identification of the closest matches between fundamental spectral features in the measured spectra and the scaled predicted data.

The quantitative results from spectral matching led to the generation of spectral barcodes that represent the measured fundamental signatures of specific species and the calculated features of potential structures across various theoretical levels. These barcodes enable the computation of average Euclidean and Manhattan distances, as well as optimal transport distances, to rank the similarities between a specific measured conformer and candidate structures, ultimately identifying the best match that corresponds to the experimentally observed conformer.

To demonstrate the performance of the employed approaches, we apply them to analyze 2-phenylethyl alcohol (phenylethyl alcohol (PEAL)) in both mono- and dihydrated forms,^45^ 2-(2-fluorophenyl)­ethyl alcohol (2-FPEAL) conformers,^46^ and 2-(2-fluorophenyl)-ethylamine (2-FPEA) monohydrate,^41^ see Figure S2 of the Supporting Information. This evaluation provides a rigorous test of the spectral matching methods. By using Euclidean/Manhattan distances and Kuhn-Munkres assignment, we achieve the highest predictive accuracy, highlighting the ability of the proposed methodology to differentiate the spectra of closely related structures.

## Results and Discussion

### Database and Scaling Factors

Considering that the observed features in the vibrational spectra are *Q*-branches with a relatively low bandwidth and that rotational fine structures could not be resolved due to limited spectral resolution, we used the band maxima as estimated fundamental frequencies. The use of these maxima is sufficient for direct comparison with calculated harmonic frequencies.

Spectral features were selected through careful visual inspection, supported by vibrational calculations to ensure their consistency. Only distinct peaks with a signal-to-noise ratio >2.5 and full-width at half-maximum (fwhm) > 4 cm^–1^ were included, to exclude noise spikes and spurious features. Based on these fundamentals and their intensities, we compiled a database, Supporting Information Data S1.

The database contains 824 jet-cooled loss- and gain- *Q*-branches of neurotransmitter conformers, analogs, and their hydrates.^34,35,38–46^ The number of features varies for each conformer due to factors like population in the molecular beam and overlap of loss lines of a specific conformer with gain lines from another conformer. For example, only eight spectral features are identified for the A2 conformer of 2-(4-fluorophenyl)-ethylamine (4-FPEA) because of significant overlaps with ionization gain lines from the most stable conformer, indicating a low A2 population and challenging ILSR spectrum measurement. Additionally, the database includes harmonic frequencies we calculate for the corresponding species by selected DFT levels [M06-2X­(-D3), B3LYP­(-D3), and ωB97X-D] ^51–54^ with 6-311++G­(d,p) and cc-pVTZ basis sets,^55–57^ and D and D3 dispersion corrections.^58,59^

While there are comprehensive benchmarking studies evaluating the performance of density functional approximations,^60,61^ and basis sets^62^ across thermochemistry, kinetics, noncovalent interactions, barrier heights, and isomerization energies, our scope is more focused, referring to our database and a small number of methods. We selected M06-2X­(-D3), B3LYP­(-D3), and ωB97X-D^50–53^ because they are widely used and have proven effective for vibrational frequency calculations. Upon pairing them with appropriate basis sets, they offer a favorable balance between computational efficiency and accuracy. Their reliability is further supported by the availability of scaling factors derived from benchmark data sets, making them well suited for vibrational spectral analysis.

Our database, though limited to NTs, analogs, and hydrates, consists of various groups and supports the retrieval of scaling factors across different spectral ranges, including global (full spectral range), mode-dependent [O–H, N–H, C–H_*ring*_, C–H_*ethyl*_, mid- (1000–2000 cm^–1^), and low-frequencies (<1000 cm^–1^)] and range-dependent [high- (>2000 cm^–1^), mid-, and low-frequencies].^14,16–20,63^ The O–H, N–H, and C–H vibrations refer to pure stretching modes that are well-isolated and exhibit minimal coupling with other vibrational motions, allowing for mode-dependent scaling factors retrieval. Also, hydrogen bonding interactions influence most frequencies of these modes.

The collective motions in mid- and low-frequency ranges involve vibrations of large parts of the molecules, making a unique group classification challenging. Although a 1000 cm^–1^ threshold may seem arbitrary, it has been considered as an optimal cutoff,^64^ and applied in previous studies for calculating low-frequency scaling factors, supporting the validity of this approach.^16,19,20^ Thus, we adopt the previously used spectral regions for determining range-dependent scaling factors.^14,16^ While this approach improves accuracy for numerous molecular systems, further research is required to evaluate its effectiveness for complex structures. Global scaling factors, commonly used for converting calculated harmonic frequencies into experimental fundamental frequencies, are widely adopted but generally less accurate. In contrast, range-dependent scaling factors—though more precise—are rarely used and limited to specific methods and frequency ranges.^16–18,20,64^ Mode-dependent scaling factors offer even greater specificity but have so far only been reported for select cases like C–O stretching.^65,66^

It is worth noting that anharmonicities affect both the energies and spectral intensities of fundamental vibrations, as shown by VPT2 results.^67,68^ Determining intensity scaling factors is required and would be helpful, but this work focuses only on frequency scaling factors due to their greater accuracy. Comparing experimental and calculated Raman intensities is challenging because of Placzek’s polarizability theory limitations and experimental uncertainties.^69^ Retrieving intensity scaling factors from measured ILSRS compared with calculated harmonic intensities is even more challenging and remains an issue to be addressed later.

We derived scaling factors (λ) and their associated uncertainties (Δλ), as shown in Table 1, by visually comparing the measured fundamental bands in spectra with the calculated vibrational spectra. These scaling factors were determined by comparing experimental fundamental frequencies, ν_*i*_, with calculated harmonic, *ω*_*i*_, frequencies. We evaluated scaling accuracy^17–19^ by calculating minimal and maximal frequency differences (error bars) along with statistical measures, including root-mean-square (RMS), mean absolute deviation, and standard deviation (σ), marked by rhombus, circle, and square, respectively, Figure 1. These metrics enable the comparison of scaled harmonic and experimental fundamental frequencies.

**Table 1:** Derived Frequency Scaling Factors and Uncertainties (in Parentheses) at Various Theoretical Levels

Scaling factor/Theory level	global	O–H	N–H	C–H*ring*	C–H*ethyl*	mid	low	high
M06-2X/6-311++G(d,p)	0.9523 (108)	0.9402 (31)	0.9454 (31)	0.9533 (20)	0.9493 (52)	0.9735 (83)	0.9727 (107)	0.9486 (57)
M06-2X-D3/6-311++G(d,p)	0.9523 (108)	0.9402 (30)	0.9454 (31)	0.9533 (21)	0.9493 (52)	0.9735 (82)	0.9728 (107)	0.9486 (57)
M06-2X-D3/cc-pVTZ	0.9534 (94)	0.9454 (38)	0.9485 (28)	0.9535 (21)	0.9499 (51)	0.9723 (83)	0.9672 (112)	0.9502 (46)
B3LYP/6-311++G(d,p)	0.9648 (98)	0.9583 (61)	0.9564 (26)	0.9640 (22)	0.9631 (55)	0.9833 (62)	0.9837 (123)	0.9615 (53)
B3LYP-D3/6-311++G(d,p)	0.9649 (93)	0.9599 (74)	0.9566 (25)	0.9642 (21)	0.9634 (55)	0.9818 (65)	0.9790 (134|)	0.9619 (54)
B3LYP-D3/cc-pVTZ	0.9653 (79)	0.9641 (79)	0.9590 (22)	0.9637 (21)	0.9640 (56)	0.9790 (58)	0.9731 (147)	0.9630 (50)
ωB97X-D/6-311++G(d,p)	0.9541 (109)	0.9399 (67)	0.9435 (35)	0.9566 (27)	0.9543 (56)	0.9712 (8)	0.9699 (15)	0.9510 (79)
ωB97X-D/cc-pVTZ	0.9539 (90)	0.9435 (65)	0.9453 (23)	0.9556 (22)	0.9537 (54)	0.9688 (66)	0.9655 (115)	0.9513 (65)

**Figure 1:**
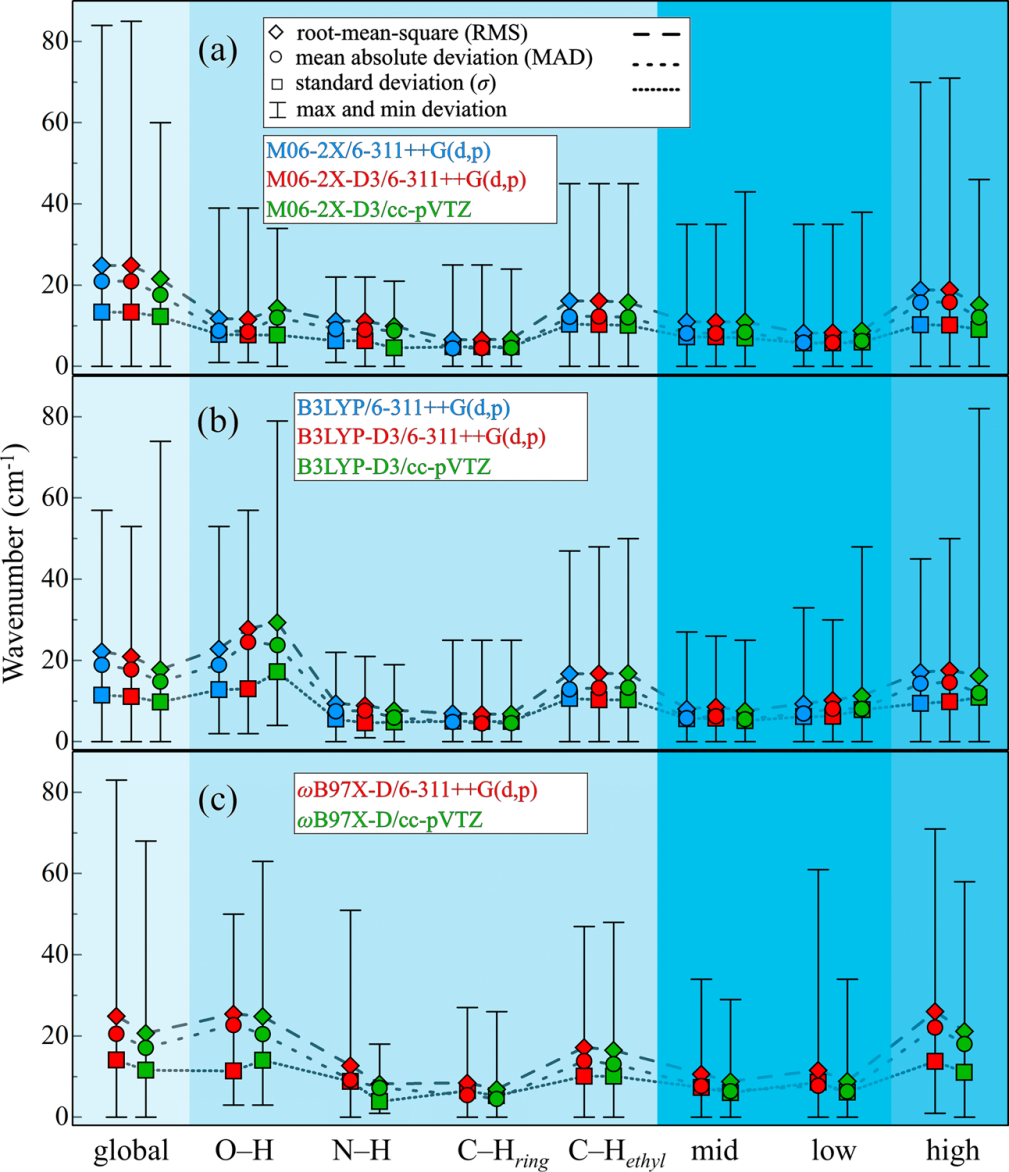
Different frequency measures between scaled harmonic and fundamental frequencies for global and mode- and range-dependent scaling factors appear in the blue-shaded sections. The right section includes high-, mid-, and low-frequency regions, where the last two also participate in the mode-dependent scaling factors.

Figure 1 shows that the global scaling factors and statistical measures for M06-2X and B3LYP without and with dispersion corrections (D3) using the 6-311++G­(d,p) basis set yield close values, while the cc-pVTZ basis set improves accuracy. This preservation trend also occurs for the ωB97X-D functional with the two basis sets. Comparisons of our derived global scaling factors with those from other databases (Table 2) show no significant discrepancies, with differences within absolute uncertainties, indicating the reliability of our scaling factors. The stability of the scaling factors was confirmed by recalculating them using only the top 20% of the most intense experimental fundamental features. The results showed minimal variations and stayed within the uncertainty margins of the full data set, demonstrating consistent derivation.

**Table 2:** Comparative Global Scaling Factors (λ) and Uncertainties (Δλ) for Different Levels of Theory and Benchmark Data Sets

M06-2X		B3LYP		ωB97X-D	
6-311++G(d,p)	cc-pVTZ	6-311++G(d,p)	cc-pVTZ	6-311++G(d,p)	cc-pVTZ
0.9523 ± 0.0108^a^	0.9534 ± 0.0094^a^	0.9644 ± 0.0091^a^	0.9653 ± 0.0079^a^	0.9539 ± 0.0090^a^	0.9545 ± 0.0098^a^
0.944^b^	0.948 ± 0.027^c^	0.959^b^	0.965 ± 0.020^c^		0.956^e^
0.947 ± 0.028^c^	0.955^e^	0.963 ± 0.020^c^	0.967^e^		
0.9567 ± 0.0371^d^			0.9691^f^		

a This work.

b ref 70.

c ref 71.

d ref 72.

e ref 68.

f ref 64.

Also, mode-dependent scaling factors demonstrate superior performance compared with global and range-dependent ones. For instance, using M06-2X and M06-2X­(-D3) with 6-311++G­(d,p) basis reduces errors by ∼50% relative to global scaling factors, except in the case of C–H_*ethyl*_, which exhibit larger deviations. These discrepancies are likely due to anharmonic effects, such as Fermi resonance, which are not captured by scaling factors. Such interactions can shift apparent frequencies, alter intensities, and complicate direct comparisons to harmonic predictions. Among the tested methods, M06-2X­(-D3)/6-311++G­(d,p) shows a better performance. Our observations align with those of Zapata Trujillo and McKemmish,^20,63^ who evaluated both range-dependent and global scaling approaches and recommended the former for improved frequency predictions.

It is interesting to note that variations between global and specific discrepancies arise due to differences in vibrational frequencies and the number of vibrations in each subgroup. Collective modes in mid- and low-frequency ranges have a stronger influence on global scaling, making it less suitable for higher-frequency stretching modes. This observation supports the use of mode-dependent scaling factors.

In addition, the scatterplots of the frequencies demonstrate a strong correlation between mode-dependent scaled harmonic frequencies and measured fundamental ionization-loss- and -gain SRS frequencies, with Pearson correlation close to one [e.g., 0.99994 for M06-2X-D3/6-311++G­(d,p)], see Figure 2, and slightly less for other theory levels. This highlights the utility of our database for benchmarking, particularly given the limited availability of scaling factors for various methods and basis sets. However, this database is specific to neurotransmitters and their analogs and hydrates, lacking the representation of all functional groups.

**Figure 2:**
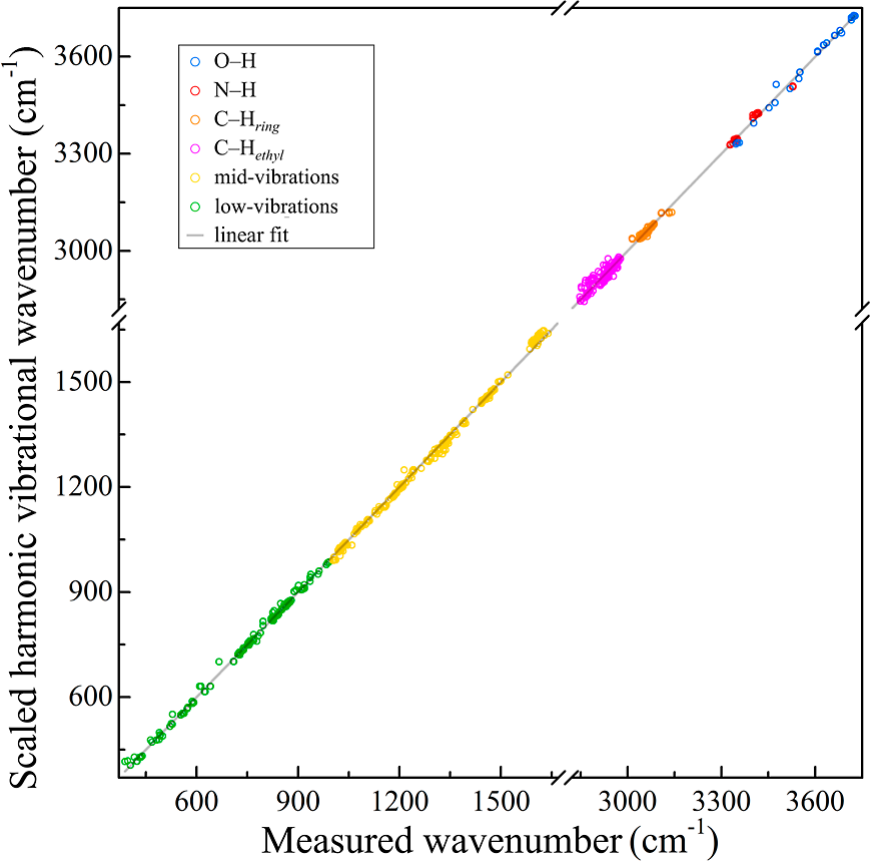
Scattered plot of mode-dependent scaled harmonic frequencies against measured fundamental ionization-detected stimulated Raman frequencies and the resulting fitting line. The scaled frequencies are at the M06-2X-D3/6-311++G­(d,p) level of theory.

It is worth noting that mode-dependent scaling factors also suffer from significant drawbacks, primarily poor portability, due to their higher specificity. Since these factors are tailored to individual vibrational modes within a given system, their general applicability remains to be tested for different molecules, limiting their adoption. Additionally, they can obscure deficiencies in the underlying electronic structure method. If scaling factors are applied when high accuracy is not the primary goal, then global scaling factors may provide a practical and more transformable compromise between simplicity and reliability.

To further assess the reliability of the scaling approaches, we analyzed the distribution of prediction errors,^20^ defined as the differences between scaled and experimental fundamental frequencies, across various theoretical levels and scaling schemes. We computed the mean absolute percentage error (MAPE; see Methods in Supporting Information) as a normalized metric of accuracy. Additionally, we calculated the first (Q1), second; median (Q2), and third (Q3) quartiles for each classification, representing the 25%, 50%, and 75% of the error distribution, respectively. These metrics were obtained from 100 cycles of 70%/30% training/test partitioning, with results averaged across runs and standard deviations calculated accordingly. The average scaling factors, MAPE, and quartile values obtained for each data classification, are presented in Table 3.

**Table 3:** Scaling Factors, MAPE, and First (Q1), Second; Median (Q2), and Third (Q3) Quartiles of the Error Distribution, Calculated Over the Full Dataset^a^ ^b^

Theory level/Scaling factors type		M06-2X/6-311++G(d,p)	M06-2X-D3/6-311++G(d,p)	M06-2X-D3/cc-pVTZ	B3LYP/6-311++G(d,p)	B3LYP-D3/6-311++G(d,p)	B3LYP-D3/cc-pVTZ	ωB97X-D/6-311++G(d,p)	ωB97X-D/cc-pVTZ
Global {824}	global scaling factor	0.9523(4)	0.9523(3)	0.9534(3)	0.9649(3)	0.9650(3)	0.9654(3)	0.9541(5)	0.9539(4)
	MAPE (%)	1.57(5)	1.57(5)	1.32(4)	1.43(5)	1.31(4)	1.07(3)	1.44(4)	1.19(3)
	Q1 (cm^–1^)	9.7(7)	9.6(8)	7.5(5)	9.7(9)	9.0(7)	7.5(4)	11.2(10)	8.4(5)
	Q2 (cm^–1^)	20.1(6)	20.1(6)	15.4(8)	19.4(5)	17.4(5)	13.7(4)	17.9(7)	15.1(6)
	Q3 (cm^–1^)	31.1(7)	31.1(7)	27.4(8)	25.9(7)	24.8(6)	20.9(5)	27.1(4)	23.4(4)
Mode-dependent {O–H [34] + N–H [54] + C–H_*ring*_ [114] + C–H_*ethyl*_ [108] + mid [309] + low [175] = 824}	O–H scaling factor	0.9403(3)	0.9402(3)	0.9455(4)	0.9584(7)	0.9598(8)	0.9642(9)	0.9400(7)	0.9437(7)
	N–H scaling factor	0.9454(2)	0.9454(3)	0.9485(2)	0.9565(2)	0.9566(3)	0.9590(2)	0.9434(3)	0.9453(2)
	C–H_*ring*_ scaling factor	0.9533(1)	0.9533(1)	0.9535(1)	0.9640(1)	0.9642(1)	0.9637(2)	0.9566(2)	0.9556(1)
	C–H_*ethyl*_ scaling factor	0.9493(3)	0.9494(3)	0.9499(3)	0.9631(3)	0.9634(3)	0.9640(3)	0.9543(3)	0.9537(3)
	mid-range scaling factor	0.9735(6)	0.9735(7)	0.9723(5)	0.9833(4)	0.9818(5)	0.9791(4)	0.9712(7)	0.9688(6)
	low-range scaling factor	0.9725(24)	0.9729(21)	0.9671(19)	0.9837(25)	0.9791(23)	0.9731(24)	0.9700(19)	0.9659(22)
	MAPE (%)	0.62(3)	0.62(2)	0.65(2)	0.59(3)	0.64(3)	0.60(3)	0.67(3)	0.58(2)
	Q1 (cm^–1^)	2.6(3)	2.5(3)	3.0(2)	2.3(4)	2.7(3)	2.2(2)	2.6(5)	2.3(3)
	Q2 (cm^–1^)	5.5(6)	5.4(5)	6.0(4)	5.0(6)	5.7(6)	5.1(5)	5.5(6)	5.2(4)
	Q3 (cm^–1^)	10.7(7)	10.5(6)	10.8(5)	9.9(6)	10.6(6)	9.9(5)	12.3(7)	9.7(6)
Range-dependent {high [340] + mid [309] + low [175] = 824}	high-range scaling factor	0.9486(2)	0.9486(2)	0.9502(1)	0.9615(2)	0.9619(2)	0.9630(2)	0.9510(3)	0.9513(2)
	mid-range scaling factor	0.9735(6)	0.9735(7)	0.9723(5)	0.9833(4)	0.9818(5)	0.9791(4)	0.9712(7)	0.9688(6)
	low-range scaling factor	0.9725(24)	0.9729(21)	0.9671(19)	0.9837(25)	0.9791(23)	0.9731(24)	0.9700(19)	0.9659(22)
	MAPE (%)	0.68(3)	0.67(2)	0.67(2)	0.62(3)	0.67(3)	0.61(2)	0.76(3)	0.64(2)
	Q1 (cm^–1^)	3.2(4)	3.2(4)	3.3(2)	2.8(4)	3.2(4)	2.4(2)	3.3(6)	2.8(3)
	Q2 (cm^–1^)	6.7(7)	6.6(6)	6.9(5)	5.9(7)	6.6(6)	5.4(6)	7.0(6)	6.4(5)
	Q3 (cm^–1^)	14.4(7)	14.3(8)	12.1(6)	12.1(6)	12.7(6)	10.8(5)	17.6(8)	14.2(5)

a Scaling factors are categorized as global, range-dependent, or mode-dependent and are specified for each subgroup within the dataset (as indicated in braces). Values in parentheses denote the standard deviation in the last digit, calculated over 100 iterations of random 70%/30% training/test splits.

b For mode- and range-related vibrations, the values in square brackets indicate the number of frequencies associated with each type, while curly braces show the total number of vibrations present in each data set used for the partitioning.

Comparison of these values reveals key trends. The mean scaling factors from randomized runs match or fall within the uncertainty bounds of the deterministic values in Table 1. Conversely, each deterministic value lies within one standard deviation of the corresponding values obtained from the randomized runs. The relatively low MAPE values across all classifications confirm that the scaling factors effectively reduce relative errors and improve predictive accuracy. Quartile statistics further characterize the error distribution, with the median (Q2) serving as a robust indicator of the typical discrepancy between scaled harmonic predictions and experimental fundamentals.^20^ Calculations using range- and mode-dependent scaling factors yield lower absolute wavenumber differences than those obtained with global scaling, with mode-dependent scaling achieving the greatest reduction. The small standard deviations from 100 iterations of random 70%/30% train–test splits confirm the stability of these results. Collectively, these findings demonstrate that improvements are systematic and robust across the data set.

### Modeling and Structure Identification

We present the process for spectral matching using the selected methods: cross-correlation and distance metrics (Euclidean, Manhattan, and optimal transport) (see below). Figure 3 shows a flowchart for evaluating similarity between features in measured and predicted spectra for different structures (see Methods in Supporting Information).

**Figure 3:**
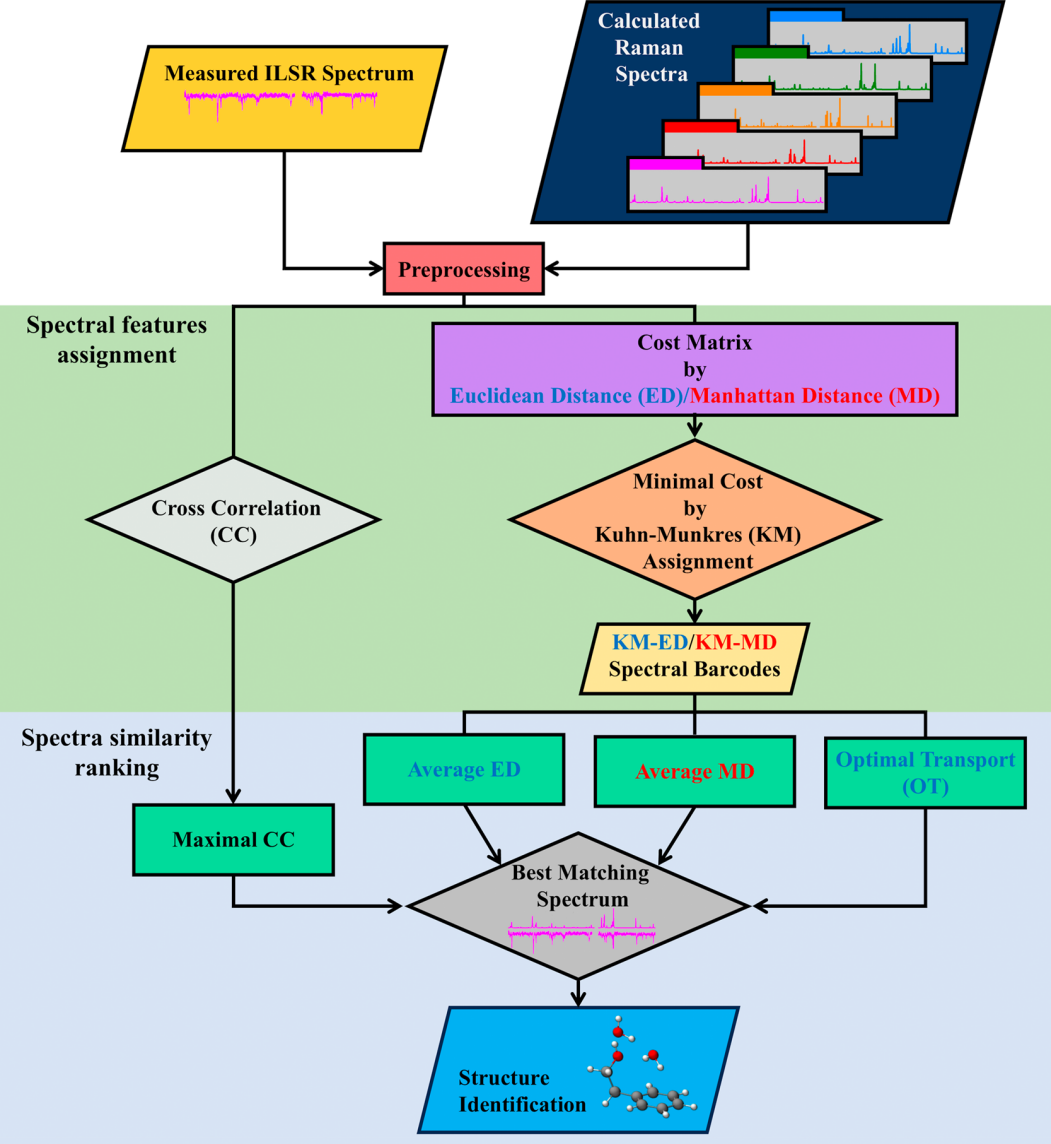
Workflow for matching measured and calculated spectra to identify structures. It involves preprocessing the spectra, calculating similarities using various methods, and finding the best match to identify a structure.

The procedure starts with preprocessing of the measured ILSR spectrum and predicted Raman signatures for various conformers. This involves normalizing the signal, scaling harmonic frequencies, and extracting representative frequencies and intensities, as shown in Figure 3. For cross-correlation, we use the entire measured spectrum to create a full spectrum vector for each computed spectrum, aligning feature intensities with corresponding indices based on frequency matching. It is important to note that scaling harmonic frequencies is essential, as comparative tests have demonstrated that unscaled frequencies often lead to inaccurate spectral features identification and, consequently, incorrect structural assignments.

In contrast, the distance metric approaches focus on discrete spectra consisting of features extracted manually from the measured data. After preprocessing, we either assess the cross-correlation of the measured spectrum and the predicted spectra of the different conformers, Figure 4a, or calculate Euclidean and Manhattan distances to generate cost matrixes, Figure 4b. The Kuhn-Munkres assignment is then applied to identify the minimal cost matrix,^49,50^ Figure 4c.

**Figure 4:**
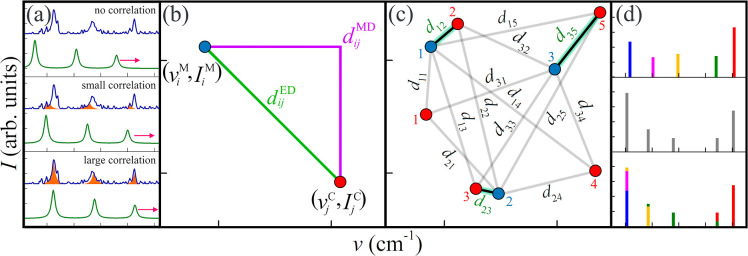
Schematic of similarity metrics utilized in spectral analysis, including: (a) cross-correlation for entire spectra,^73^ which measures similarity by displacing the calculated spectrum (green) relative to the measured one (blue), (b) Euclidean and Manhattan distances, which evaluate distances between extracted features from the measured spectrum (blue dots) and calculated spectrum (red dots), considering frequencies and weighted intensities. The resulting distances generate cost matrixes and the (c) Kuhn-Munkres algorithm assigns features to minimize the cost matrix, thereby generating spectral barcodes for specific compounds. The measured barcode is then compared to predicted barcodes of different conformers using average Euclidean and Manhattan distances to rank similarities. (d) Optimal transport classification,^48^ links the measured spectrum (top) with the calculated Euclidean distance-Kuhn-Munkres barcode (center) through an optimal transport plan (bottom) transport cost. This plan redistributes intensity using Euclidean distance for frequency alignment, ranking similarity to find the best match.

Although previous analyses, using Pearson correlation, RMS, Euclidean and Manhattan distances,^74^ focused on determining spectral matches by comparing frequency vectors solely, we chose methods that consider both frequency and intensity vectors to better capture spectral patterns. Figure 5 presents a comparison between a small portion of the measured spectrum^45^ [panel (a)] and the scaled harmonic spectrum [panel (b)] of the monohydrate, PEAL-H_2_O C*. The C* denotes the feature at 37,669 cm^–1^ in the R2PI spectrum. This feature allowed the measurement of the ILSR spectrum by setting the UV laser at this frequency and monitoring the *m*/*z* = 122 mass channel. Assigning features based just on frequency is challenging, as the calculated spectrum contains more features than the measured one, leading to problematic assignment when using only frequency-based Euclidean distance, panel (c).

**Figure 5:**
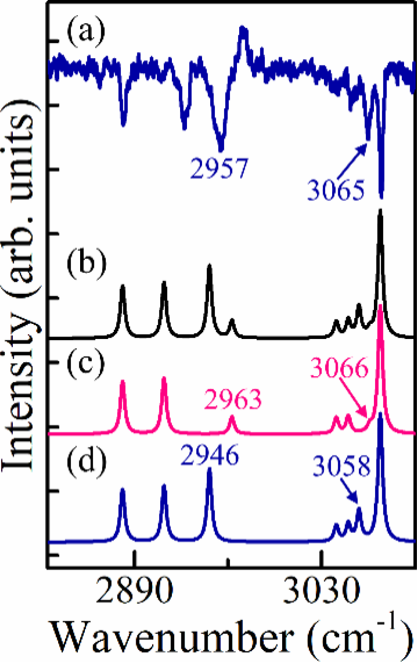
Small portions of the measured, calculated, and resulting assignments spectra. (a) Measured ionization-loss mass spectrum of 2-phenyethyl alcohol-H_2_O C* (ref 44) and (b) mode-dependent scaled harmonic Raman spectrum for the global minimum structure at the M06-2X-D3/6-311++G­(d,p) level, convolved with Lorentzian lines [full width at half-maximum of 4 cm^–1^]. (c,d) Resulting assignment from the minimal cost matrixes of the Kuhn-Munkres algorithm, using one-dimensional Euclidean distances for frequencies and two-dimensional Euclidean distances for frequencies and weighted intensities. The marked frequency values are given in cm^–1^.

In contrast, the Euclidean distance that incorporates weighted intensities [panel (d)] achieves a close match between calculated and measured spectra, enabling more reasonable assignment. Therefore, in subsequent tests, we employ the selected methods, which also account for intensities, to assign each measured feature to its corresponding calculated feature in a potential matching spectrum.

Given the choice of the similarity approaches can significantly influence model performance and prediction accuracy, we will evaluate the selected methods, including cross-correlation, Euclidean, Manhattan, and optimal transport distances (Figure 4a,b,d). Optimal transport^48^ calculates the cost of transforming intensities from their positions in the measured spectrum (top panel, Figure 4d) to those generating the Raman spectrum (central panel, Figure 4d) (see Methods in Supporting Information). This cost is determined by minimizing the work required to transfer intensity units from the features in the measured spectrum to those forming the bottom spectrum, as shown in Figure 4d. The process accounts for the Manhattan distance along the frequency axis required to transport intensity between features.

We investigate the potential to associate a measured ILSR spectrum with a specific structure by analyzing single and double PEAL hydrates^45^ and 2-FPEAL^46^ conformers, using the M06-2X-D3/6-311++G­(d,p) theory level. These systems are suitable for testing our approach due to the diverse three-dimensional structures that they form. These variations arise from side chain folding and the formation of numerous intra- and intermolecular hydrogen bonds,^45,46^ leading to slight differences in energies and spectra. Supporting Information Figure S3A–C show the shapes of PEAL-H_2_O, PEAL-(H_2_O)_2_, and 2-FPEAL, ranked by relative energies, including zero-point vibrational energy corrections, following full structural optimizations and zero-point vibrational energy corrections at the M06-2X-D3/6-311++G­(d,p) level. While energy rankings might differ at other theory levels,^46^ our goal here is to match measured spectra with calculated spectra to determine species structures, Supporting Information Figures S4.

Our approach was evaluated by plotting cross-correlations and distance values using the Kuhn-Munkres assignment with Euclidean, Manhattan, and optimal transport distance metrics. Figure 6a–c show results for PEAL-H_2_O C* and F, PEAL-(H_2_O)_2_ C* and G, and 2-FPEAL G1h and A1f with their geometries in Figures S2 and S3 of the Supporting Information. The nomenclature used for the identified geometries is consistent with refs 45 and 46 .

**Figure 6:**
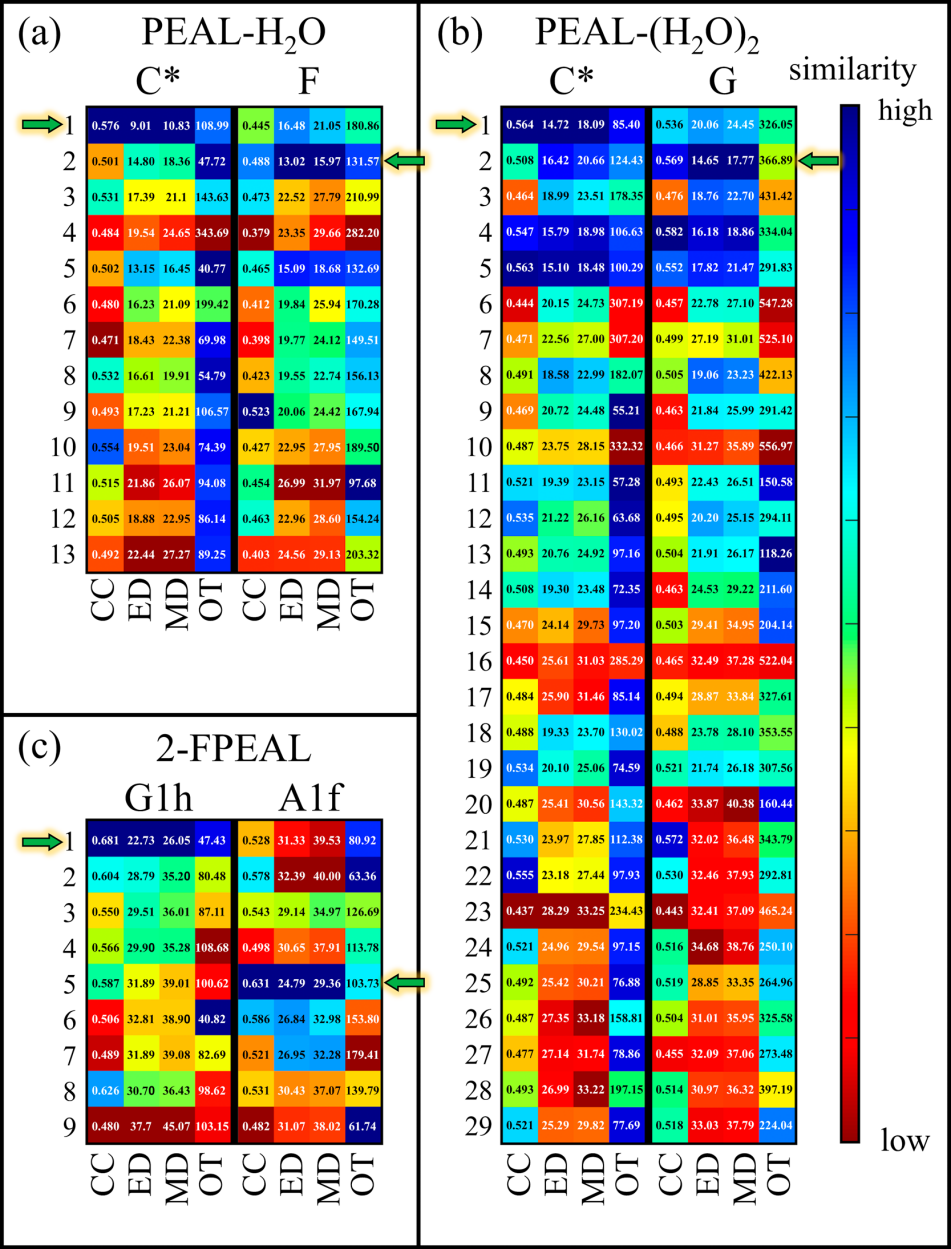
Cross-correlations and distance metrics representing similarities between mode-dependent scaled Raman spectra at the M06-2X-D3/6-311++G­(d,p) level and experimental ionization-loss stimulated Raman signatures of specific species. These values are derived from the optimized geometries and their corresponding spectra as shown in Figures S3 and S4 of the Supporting Information. The color maps show results for 2-phenylethyl alcohol (PEAL) (a) mono- and (b) dihydrates, and of (c) 2-(2-fluorophenyl)­ethyl alcohol (2-FPEAL) conformers. These maps use cross-correlation and Kuhn-Munkres assignment, including average Euclidean and Manhattan distances and optimal transport distances. Within each cell, cross-correlation values and distances are visualized using gradients with color bar on the right indicating the best matches. The horizontal arrows mark the structures identified through visual comparisons.

As shown by the right color bar in Figure 6, where blue denotes maximal similarities and red denotes minimal similarities for each method, it is probable that the highest cross-correlations and lowest distances, indicated by the bluest color, should identify species structures. Using these criteria for conformers identification, we find that the evaluated Euclidean and Manhattan distances and mostly cross-correlations (except for PEAL-(H_2_O)_2_ G), correspond closely with those determined through visual comparisons (indicated by horizontal arrows) of measured and calculated spectra, effectively identifying the structures.^45,46^ In contrast, optimal transport fails at the structural identification of the species. Supporting Information Data 2 shows that visual matching of predicted data at the M06-2X-D3/6-311++G­(d,p) level of theory strongly agrees with the quantitatively assigned data at the same level.

It is important to note that the uncertainties in the Euclidean and Manhattan distances, determined through error propagation (see Methods in Supporting Information, eqs S13 and S14), range from 0.10 to 0.72, depending on the specific molecule and similarity method used. Based on the metrics and their uncertainties, some configurations may present comparable Euclidean and Manhattan distances, suggesting that the introduced criterion might not be sufficient. Therefore, in cases with minimal metric changes, it is possible to consider an additional criterion that refers to the relative energies and interconversion barriers for identifying the correct structure.

Indeed, by using the similarity criterion for PEAL-H_2_O C*, PEAL-(H_2_O)_2_ C*, and 2-FPEAL G1h, the structures are ranked as 1 and correspond to the global minimum. On the other hand, PEAL-H_2_O F and PEAL-(H_2_O)_2_ G, and 2-FPEAL A1f are associated with local minima, with the hydrates ranked as 2 and the 2-FPEAL as 5. This ranking is consistent with the tendency of molecular beams to primarily contain lower-energy species. For 2-FPEAL, the low potential energy barriers separating wells on the potential energy surface allow relaxation and the observation of a higher energy conformer.^46^

These findings confirm that the cross-correlation and Euclidean/Manhattan distance-based Kuhn-Munkres assignment effectively recognize spectral data patterns through similarity evaluations. Particularly, these methods and their subsequent outcome enable informative determinations matching measured to closely related calculated spectra while distinguishing dissimilar ones. Conversely the optimal transport approach, which may redistribute feature intensity to additional ones, proves ineffective for identifying similarities between measured and calculated spectra.

Nevertheless, it is important to note that our structural identification of the PEAL hydrates and 2-FPEAL conformers using the Euclidean/Manhattan distance-based-Kuhn-Munkres assignment differs from previous assignments based on low- and high-resolution electronic spectroscopy.^75,76^ For the PEAL hydrates,^75^ they attributed the C* R2PI feature to the global minimum structure, PEAL-H_2_O C*, consistent with our findings but without recognizing the contribution of PEAL-(H_2_O)_2_ C*. Interestingly, they could not resolve the F band due to its low intensity, while the G band, despite its higher intensity, appeared superimposed on a high background, exhibiting a nonresolved rotational microstructure, precluding its rotational fit. Therefore, they tentatively assigned this band to a structure with a sideways-bound water molecule rather than the PEAL-(H_2_O)_2_ G structure, identified in our study.^45^ Similarly, for 2-FPEAL, while our identification of the global minimum structure agreed with theirs, the lack of a convincing fit of the rotational structure for the other band led them to tentatively assign it to the G1f conformer. In contrast, our findings, supported by visual comparison^46^ as well as cross-correlation and Euclidean/Manhattan distance-based Kuhn-Munkres assignment, point to the A1f conformer as the correct assignment.

While the Euclidean and Manhattan distance metrics effectively identify structurally isolated conformers using mass-resolved ILSR spectra in comparison to predicted spectra, several factors can influence their success. For example, experimental systems may not provide isolated spectra or adequate spectral resolution, leading to overlapping spectral features and noise that can complicate the analysis. Additionally, computational predictions depend on the selected theoretical model and basis set, which may result in discrepancies in predicted vibrational frequencies or intensities due to harmonic approximations and inaccuracies in polarizabilities. These factors affect the alignment between measured and calculated spectra.

Our current methodology does not address some of these issues, including overlapping measured spectral features and computational errors. To enhance robustness, incorporating deconvolution methods, data preprocessing techniques, and error-correcting protocols could be beneficial. Extended case studies with complex data sets could further refine the metrics and broaden their applicability across diverse chemical systems.

## Summary

We have developed a new database from our previously measured broad-range ionization-loss and -gain SRS spectra of compounds containing phenyl or indole rings attached to ethylamino (or ethyl alcohol) side chains along with their hydrates. These compounds exhibit high structural complexity due to their intricate conformational landscapes. By comparing the experimental fundamental bands with the predicted Raman spectra of visually identified structures across different levels of theory, we derived global, range-dependent, and mode-dependent scaling factors. The global scaling factors are close to those previously reported for similar theoretical levels, affirming the reliability of our database. Statistical analyses indicate that mode- and range-dependent scaling factors outperform global scaling approaches, showing higher accuracy and offering promising applications for benchmarking future predictions.

Further testing of our approach involves assigning measured ILSR spectra that reflect isolated individual structures by selective ion detection through various mass channels. We use cross-correlation for the entire spectra, Euclidean/Manhattan distance-Kuhn-Munkres assignment to generate spectral barcodes, and determine their average Euclidean/Manhattan or optimal transport distances obtained via comparison of measured spectra to mode-dependent scaled spectra. This strategy demonstrated that both cross-correlation and, even more effectively, the Euclidean/Manhattan distance-based Kuhn-Munkres approaches successfully capture spectral data patterns by evaluating similarities through these metrics, providing a basis for spectral rankings. These evaluations enable matching measured with closely related calculated spectra, informative structural identifications, and classification of unrelated ones. These findings represent a new frontier of spectral identifications, and we anticipate a new window opening to structural recognition.

## Supplementary Material











## Notes

Schematics of ionization-loss and -gain stimulated Raman spectroscopies, geometries and labels of conformers and hydrates of the various species, and Methods; geometries of global and local minima of conformers of 2-phenylethyl alcohol mono- and dihydrates and of 2-(2-fluorophenyl)­ethyl alcohol; measured ionization-loss stimulated Raman spectra pointing downward and scaled harmonic Raman spectra upward of these conformers; and spreadsheets of measured and calculated features for different compounds and Kuhn-Munkres-Euclidean distances and visual spectral assignments (PDF)
(XLSX)
(XLSX)
(TXT)
(TXT)
