# Supplementary material for: Analyzing Spectral Similarities for Structural Identification Using a New Benchmark Database
Source: J Phys Chem A. 2026 Jan 12;130(3):602–13. doi: 10.1021/acs.jpca.5c06253 (PMC12833857; doi:10.1021/acs.jpca.5c06253)
Supplement: Supplementary file 1 [file jp5c06253_si_001.pdf]

Supporting Information

Analyzing Spectral Similarities for Structural Identification using a New Benchmark Database

Rami Rahimi, Noga Saban, and Ilana Bar*

Department of Physics, Ben-Gurion University of the Negev, Beer-Sheva 8410501, Israel

Contents

Figure S1. a) Schematic of ionization-loss stimulated Raman spectroscopy and (b) ionization-gain stimulated Raman spectroscopy………………..………………………………………S3

Figure S2. Geometries and labels of measured conformers and hydrates of the various species. The database in Data 1 includes the features of these species………………………………………..S4

METHODS……………………………………………………………………………………S6

Database and determination of scaling factors……………………………………………… S6

Spectral similarities and structural identifications……………………………………………S10

References……………………………………………………………………………… …..S14

Figure S3. Geometries of the global and local minima of conformers of 2-phenylethyl alcohol (PEAL) (A) mono- and (B) di-hydrates, and (C) 2-(2-fluorophenyl)ethyl alcohol in the ground electronic state, S_0, as determined following full structural optimizations at the M06-2X-D3/6-311++G(d,p) level of theory with zero-point vibrational energy corrections...S20

Figure S4. Measured ionization-loss stimulated Raman spectra pointing downward and scaled harmonic Raman spectra (at the M06-2X-D3/6-311++G(d,p) level of theory) upward for conformers of 2-phenylethyl alcohol (PEAL) (A) mono- and (B) di-hydrates, and (C) 2-(2-fluorophenyl)ethyl alcohol...S23

Data 1. Database including the conformers and mass-to-charge ratio (*m*/*z*) used for our measurements of ionization-loss and -gain stimulated Raman features (frequencies and normalized intensities) and corresponding visually matched calculated harmonic frequencies at different levels of theory [M06-2X/6-311++G(d,p), M06-2X-D3/6-311++G(d,p), M06-2X-D3/cc-pVTZ, B3LYP/6-311++G(d,p), B3LYP-D3/6-311++G(d,p), B3LYP-D3/cc-pVTZ, *ω*B97X-D/6-311++G(d,p), and *ω*B97X-D/cc-pVTZ] computed from the resultant optimized structures, mode types or frequency ranges, and links to the source articles of the measurements…………………………………………………………………………..S25

Data 2. Database including the conformers and mass-to-charge ratio (*m*/*z*) for measurements of ionization-loss stimulated Raman features (frequencies and normalized intensities) of 2-phenylethyl alcohol (PEAL) mono- and di-hydrates, and 2-(2-fluorophenyl)ethyl alcohol and corresponding quantitatively and visually matched Raman signatures (frequencies and normalized intensities) calculated at the M06-2X-D3/6-311++G(d,p) level of theory. The quantitative matching results from calculation of Euclidean and Manhattan distances, implementation of the

Kuhn-Munkers algorithm and computation of the average Euclidean/Manhattan distances between frequencies and intensities of measured and calculated spectra……………………………………………...……………………………...…S25

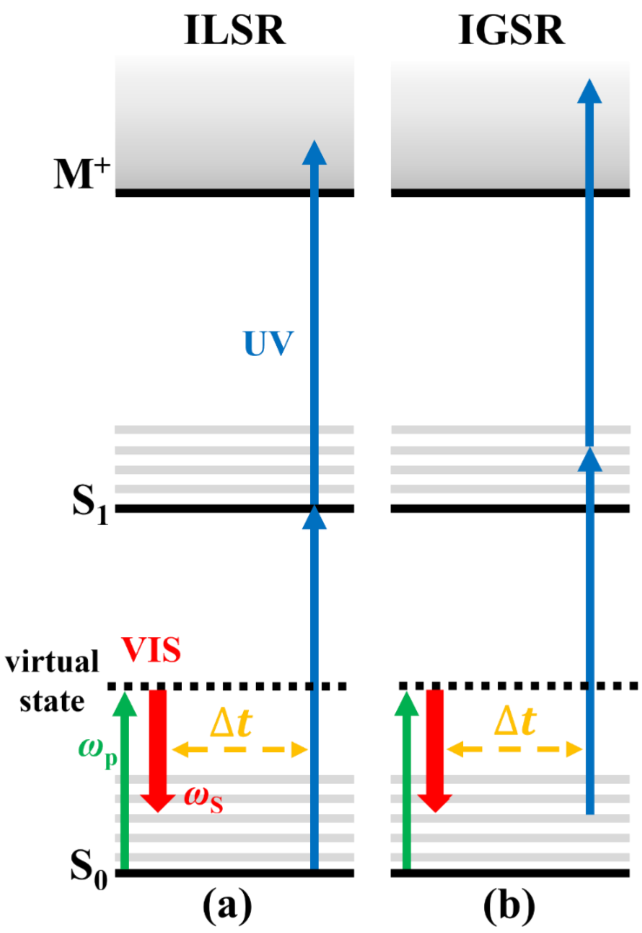

Figure S1. (a) Schematic of ionization-loss stimulated Raman spectroscopy (ILSRS), and (b) ionization-gain stimulated Raman spectroscopy (IGSRS) spectroscopy. The visible (VIS) photons (pump, ω_P, and tunable Stokes, ω_S) induce stimulated Raman scattering, while the ultraviolet (UV) photons probe the vibrational ground or excited states via one-color resonant two-photon ionization (R2PI). The Δt represents the delay between the exciting and probing lasers.

2-(2-fluoro-phenyl)ethylamine (2-FPEA)

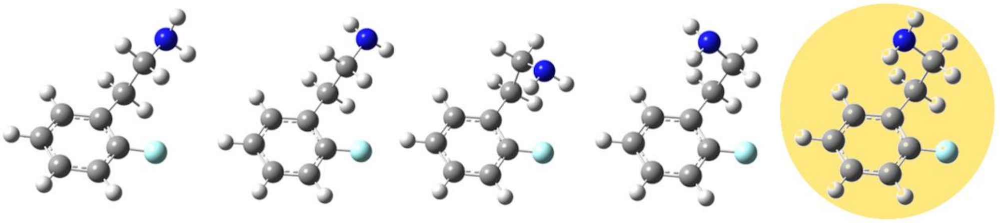

A1h A2 G2f G2h G1h

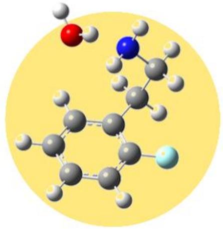

2-FPEA-H_2O

2-(4-fluoro-phenyl)ethylamine (4-FPEA)

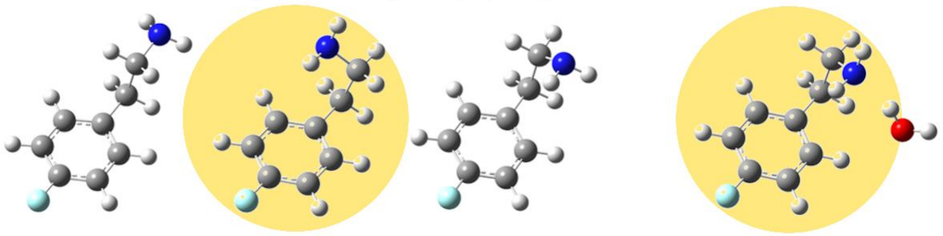

A2 G1 G2 4-FPEA-H_2O

2-phenylethlamine (PEA)

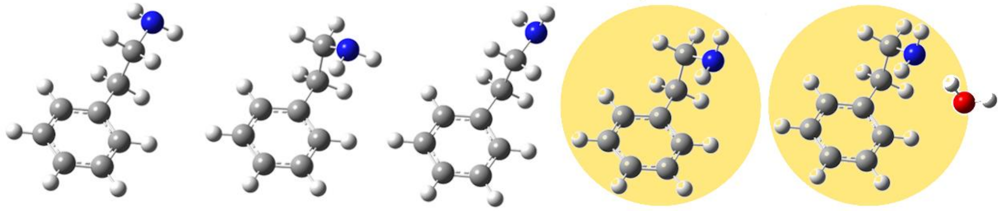

A B D C PEA-H_2O

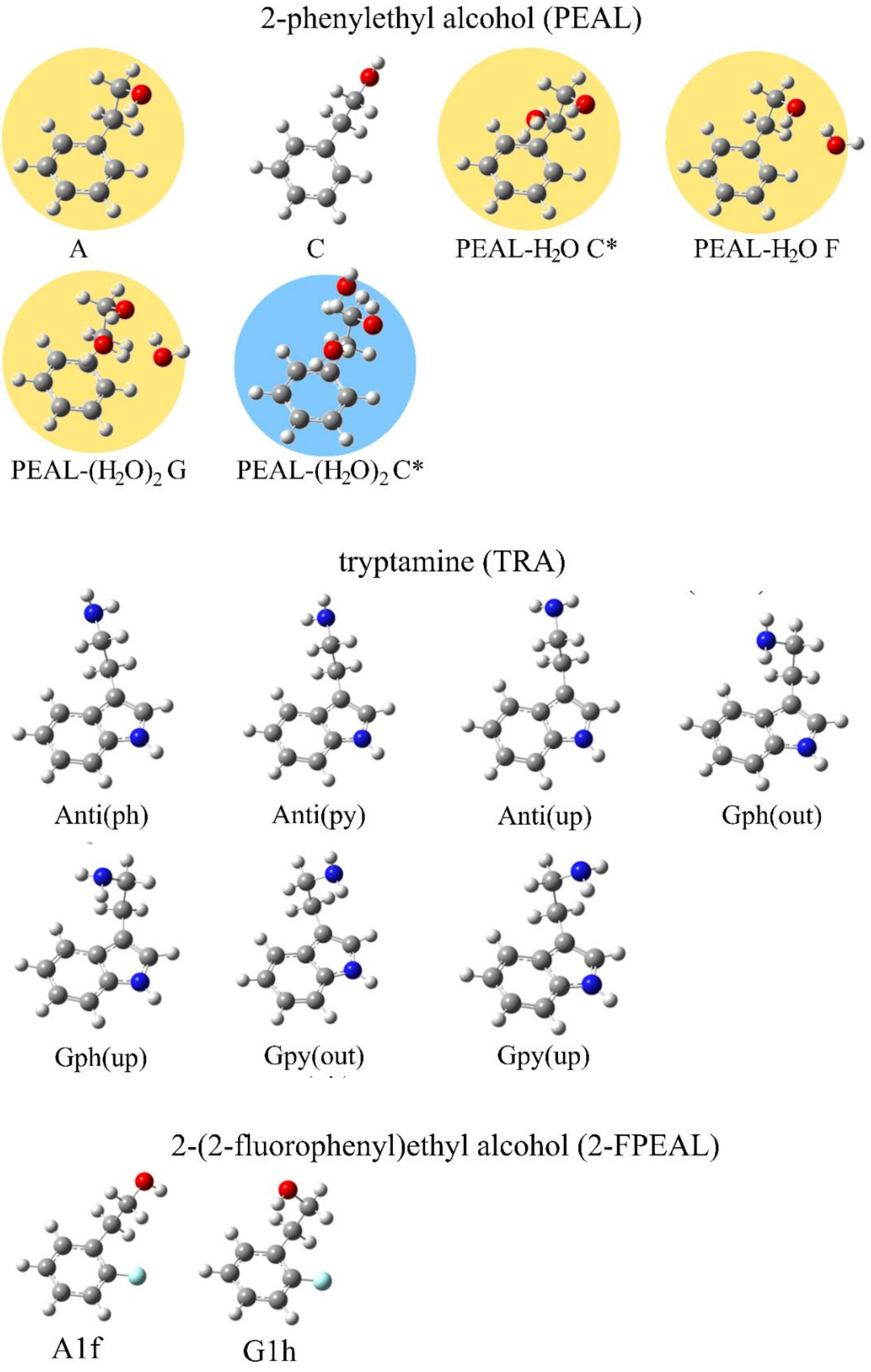

Figure S2. Geometries and labels of measured conformers and hydrates of the various species, with yellow circles highlighting the most stable *gauche* configurations of the monomer in hydrates. For PEAL-(H_2O)$_2$, the blue circle shows a shift to *trans-gauche* PEAL after double hydration.

■ METHODS

Database and determination of scaling factors

We manually generated initial structures using chemical intuition and insights from previously studied related systems. A thorough optimization process was conducted, beginning with the Merck molecular force field (MMFF94s) in Avogadro[1] applied to various initial structures to identify potential candidate geometries for 2-phenyethylamine (PEA),[1] 2-(4-fluorophenyl)ethylamine (4-FPEA),[2] 2-FPEA,[3] 2-phenylethyl alcohol (PEAL),[4] 2-(2-fluorophenyl)ethyl alcohol (2-FPEAL),[5] and tryptamine (TRA) conformers,[6,7] PEA, 4-FPEA, and 2-FPEA monohydrates,[8-10] and PEAL mono- and di-hydrates.[11] The different geometries of the species are then fully optimized, employing M06-2X(-D3), B3LYP(-D3), and ωB97X-D functionals with 6-311++G(d,p) and cc-pVTZ basis sets,[12-20] in the GAUSSIAN 16 package.[21] The optimizations use "very tight" self-consistent field convergence criteria and "ultrafine" integration grids. We then calculate harmonic vibrational frequencies, Raman activities, and depolarization ratios for each optimized structure using the same package. After correcting Raman activities for the parallel polarization of the SRS beams, we convert the activities to intensities[7] using the formula of Polavarapu.[22] The features are convolved with Lorentzian lines (4 cm^{-1} FWHM) to predict Raman spectra for comparison to the measured ILSR spectra.[1,5,7-9,11,23-26] The calculated harmonic frequencies also confirm true energy minima for the different conformers.

It is worth noting that the Raman intensities were computed using static activities from harmonic frequency calculations at the selected DFT levels. While dynamic polarizabilities can provide more accurate Raman intensities under certain conditions, static polarizabilities are widely used and considered reliable for the weak-field regime used in our experiments, as noted previously.[27] Since the weak-field regime is at laser intensities less than 10^{12} W/cm^2 and our

experiments are performed at two orders of magnitude less, the use of static polarizabilities is reasonable. However, calculating complete Raman spectra for medium- and large-sized molecules beyond the harmonic approximation is a major challenge in computational chemistry.[28]

Before we can determine the scaling factors and proceed with the assignment, preprocessing of measured ILSR vibrational and calculated Raman spectra is required to ensure their consistency and comparability. The processing involves systematic extraction of the vibrational frequencies and intensities associated with each discernible peak in both spectrum types. We denote the vibrational fundamental frequencies and intensities of the *i*-th vibrational peak in the measured (*M*) or calculated (*C*) spectra by (υ_i, I_i^M) and (ω_i, I_i^C), respectively. Compiling these vibrational frequencies and intensities into a unified dataset forms a comprehensive representation of vibrational signatures in the *M* and *C* matrixes:

$$M = \begin{pmatrix} \upsilon_1 & I_1^M \\ \vdots & \vdots \\ \upsilon_{n_M} & I_{n_M}^M \end{pmatrix}, C = \begin{pmatrix} \omega_1 & I_1^C \\ \vdots & \vdots \\ \omega_{n_C} & I_{n_C}^C \end{pmatrix} \tag{1}$$

Attempting to establish the database and determine the optimal scaling factors, we generate pairs of *M* and *C* for each identified structure from our previous studies. However, due to the inherent limitations in observing low-intensity and low-frequency (< 400 cm^{-1}) features, the amount of the calculated values exceeds those observed in experimental spectra. Consequently, the number of rows in *C* surpasses that of *M*, i.e., $n_C > n_M$. To reconcile this disparity and facilitate meaningful comparisons between the datasets, we visually assign n_M rows in each *C* to their counterparts in *M* for each previously measured spectrum.[2,5-9,23-26] Afterwards, the *M* and *C* intensities are normalized by the maximal value in the high-range (> 2000 cm^{-1}) and in the mid- and low-range (< 2000 cm^{-1}) of each vector individually. This normalization allows for minimizing

potential biases or discrepancies between intensity distributions of measured and calculated spectra. These processes facilitate the establishment of the novel benchmark database consisting of 824 vibrations. Each row encompasses the measured vibrations and the corresponding visually matched ones, calculated at the above-mentioned levels of theory, see Supporting Information Data 1.

Using this database, we determine the optimal global and multiple (mode- and range-dependent) scaling factors (λ) (Eq. 2) by accounting for the frequencies in the specific ranges. The scaling factors are revealed through a least squares solution to minimize the sum of N residuals (Eq. 3) within the database, where N represents its total number of rows (selected for the calculation of different types of scaling factors). These residuals, denoted as Δ_l, correspond to the l-th calculated frequency ω_l and the corresponding l-th fundamental frequency ν_l, as previously proposed.[29]

$$\lambda = \frac{\sum_{l=1}^{N} \omega_l \nu_l}{\sum_{l=1}^{N} (\omega_l)^2} \tag{2}$$

$$\Delta = \sum_{l=1}^{N} \Delta_l = \sum_{l=1}^{N} (\lambda \omega_l - \nu_l)^2 = \sum_{l=1}^{N} (\tilde{\omega}_l - \nu_l)^2 \tag{3}$$

To reduce errors in frequencies at the lower end of the frequency range (< 1000 cm^{-1}), an inverse frequency scale factor is calculated (Eq. 4) to minimize the residual in Eq. 5.[29]

$$\lambda = \frac{\sum_{l=1}^{N} \left(\frac{1}{\omega_l}\right)^2}{\sum_{l=1}^{N} \frac{1}{\omega_l \nu_l}} \tag{4}$$

$$\Delta = \sum_{l=1}^{N} \left(\frac{1}{\lambda \omega_l} - \frac{1}{\nu_l}\right)^2 \tag{5}$$

The absolute uncertainty in the scaling factors ($\delta\lambda$) is estimated using the following equation:[30]

$$\delta\lambda = \frac{\mathrm{RMS}}{\sqrt{\sum_{l=1}^{N}(\omega_l)^2}} \tag{6}$$

where RMS is defined below. The calculations of the deviations for the scaling factors include MAD (Eq. 7), RMS (Eq. 8), and σ (Eq. 9).

$$\mathrm{MAD} = \sum_{l=1}^{N}\frac{\Delta_l^{1/2}}{N} = \sum_{l=1}^{N}\frac{|\lambda\omega_l - \nu_l|}{N} \tag{7}$$

$$\mathrm{RMS} = \left(\sum_{l=1}^{N}\frac{\Delta_l}{N}\right)^{1/2} = \left(\sum_{l=1}^{N}\frac{(\lambda\omega_l - \nu_l)^2}{N}\right)^{1/2} \tag{8}$$

$$\sigma = \left(\sum_{l=1}^{N}\frac{\left(\Delta_l^{1/2} - \mathrm{MAD}\right)^2}{N}\right)^{1/2} \tag{9}$$

We evaluated the variability in the performance of the different models using global, frequency-dependent, and mode-dependent scaling factors. To quantify errors, we computed the absolute error (AE):

$$\mathrm{AE} = e_l = |\lambda\omega_l - \nu_l| \tag{10}$$

and the mean absolute percentage error (MAPE):

$$\mathrm{MAPE} = \frac{100}{N}\sum_{l=1}^{N}\frac{e_l}{\nu_l} = \frac{100}{N}\sum_{l=1}^{N}\frac{|\lambda\omega_l - \nu_l|}{\nu_l} \tag{11}$$

Additionally, we analyzed the distribution of errors using quartiles: the first quartile (Q1) represents 25 % of the absolute error set $\{e_l\}_{l=1}^{N}$, the second quartile (Q2, median) the 50 %, and the third quartile (Q3) the 75 %.

The average MAPE and quartile values, along with their standard deviations reported in Table 3, were calculated using repeated bootstrap-based random partitioning of the dataset, allocating 70 % for training (model fitting) and 30 % for testing (model evaluation) across 100 runs.

Spectral similarities and structural identifications

The structural identification involves cross-correlation (CC) and distance metrics to match measured and calculated spectra. To compute $\mathrm{CC}(\tau)$, we downsample and smooth the measured spectrum to create a vector $S(n)$, with each entry at index *n* representing intensity at the *n*-th specific frequency. The measured spectrum was binned at 1 cm^{-1} intervals, averaging intensities within each bin. Multiple peak intensities within a bin were also averaged, considering that typical vibrational linewidths > 4 cm^{-1}, and bins without features were set to zero. A Savitzky-Golay filter (2nd order, window size of 5) was used for smoothing. Also, the spectrum was normalized separately for the low/mid (< 2000 cm^{-1}) and high (> 2000 cm^{-1}) regions. Similarly, we generate a binned spectrum vector $A(n)$, for each computed conformer spectrum, ensuring all vectors have the same length L and frequency resolution (1 cm^{-1}). This enables the calculation of the cross-correlation between the two data vectors across the entire spectral range, using Eq. (12):[31]

$$\mathrm{CC}(\tau) = \sum_{n=1}^{L} S(n)A(n+\tau) \quad (12)$$

Using MATLAB's xcorr function, we calculate $\mathrm{CC}(\tau)$ by summing the product of intensities at each spectral point n while shifting the calculated spectrum relative to the measured spectrum across all lags τ, ranging from $-(L-1)$ to $(L-1)$. The xcorr function applies zero-padding for out-of-bound indices, ensuring consistent $\mathrm{CC}(\tau)$ values. This approach allows for screening all reference spectra to identify the lag with the highest $\mathrm{CC}(\tau)$, indicating the conformer with the best spectral similarity.

For distance metrics, the process of identifying structures involves generating a series of calculated vectors, C_1 to C_Z, that represent predicted vibrational features obtained from calculations for various potential structures. Each vector represents a possible best-match candidate to the *M* vector of the specific test species. Next, we scale the frequencies in each C_x vector (x ranging from *1* to *Z* – the number of tested conformers) by the corresponding mode-dependent scaling factors and define it as $\tilde{C}_x$. Then, we calculate the d^{ED} and d^{MD} $(n_M \times n_C)$ cost matrixes, where each matrix entry, d_{ij}, represents the potential assignment cost between the i-th and j-th elements in *M* and $\tilde{C}_x$, respectively. This d_{ij} cost is the two-dimensional distance between (υ_i, I_i^M) and $(\widetilde{\omega}_i^{\tilde{C}_x}, I_i^{\tilde{C}_x})$, calculated via Euclidean distance (Eq. 13) or Manhattan distance (Eq. 14), Figure 4b. The Kuhn-Munkres algorithm,[32,33] Figure 4c, is then applied to the cost matrixes to obtain the lowest assignment cost. These results lead to optimal assignment pair features between corresponding rows and, consequently, to $\tilde{C}_x^{ED}$, $\tilde{C}_x^{MD}$, and *M* spectral barcodes of calculated and measured features exhibiting the highest similarities.

$$d_{ij}^{ED} = \sqrt{\left(\upsilon_i - \widetilde{\omega}_j^{\tilde{C}_x}\right)^2 + w^2\left(I_i^M - I_j^{\tilde{C}_x}\right)^2} \tag{13}$$

$$d_{ij}^{MD} = \left|v_i - \widetilde{\omega}_j^{\tilde{C}_x}\right| + w\left|I_i^M - I_j^{\tilde{C}_x}\right| \tag{14}$$

To address the two to three orders of magnitude imbalance between normalized intensities and fundamental frequencies, a weighting factor, w, is introduced (Eqs. 13 and 14). This factor ensures that frequency and intensity differences are appropriately balanced in the calculated distance metrics. First, we calculate the cost matrix considering only fundamental frequencies (w = 0). Features are then matched based on the minimal cost matrix, and conformers are ranked accordingly. For the best-fit calculated vector ($\tilde{c}_{BF}$), identified by one-dimensional ED ranking for frequency the mean difference in vibration frequency ($\overline{\Delta\nu\imath b}$) and intensity ($\overline{\Delta\imath nt}$) between the measured and calculated vectors are computed using:

$$\overline{\Delta\nu\imath b} = \frac{\sum_{i=1}^{N}\sum_i(v_i^M - \widetilde{\omega}_i^{\tilde{c}_{BF}})}{N} \tag{15}$$

$$\overline{\Delta\imath nt} = \frac{\sum_{i=1}^{N}\sum_i(I_i^M - I_j^{\tilde{c}_{BF}})}{N} \tag{16}$$

The weighting factor is defined as the ratio of the mean vibration frequency and intensity differences:

$$w = \frac{\overline{\Delta\nu\imath b}}{\overline{\Delta\imath nt}} \tag{17}$$

The w is then incorporated into the Euclidean and Manhattan distance calculations to balance the contribution of frequency and intensity differences.

It is worth noting that while Euclidean distances assignment for frequencies only may not always lead to an exact match for all features, it provides a reliable approximation of mean frequency and intensities, suffering from variability between experimental conditions for conformers. Furthermore, we assess the weighted Kuhn-Munkres assignment for all species in

Supporting Information Data 2 and compare them to previous visual assignments to validate the effectiveness of w. The alignment between the Kuhn-Munkres and visual assignments justifies the applicability of the derived w factor, improving the spectral assignment by quantitatively balancing intensity and frequency differences. Finally, to obtain the similarities between measured and calculated spectra, the resulting average Euclidean or Manhattan distances between the corresponding rows of $\tilde{C}_x^{ED}$ or $\tilde{C}_x^{MD}$ and M are calculated and ranked for each potential structure, allowing to determine the best matching.

Additionally, we investigated the use of computational optimal transport, Figure 4d, to reveal the similarity between each $\tilde{C}_x^{ED}$ and M. In this approach, we represent each vector by a discretized bar spectrum. The positions of each bar are at respective frequencies, and their height signifies intensities. Visual representations of these M and $\tilde{C}_x^{ED}$ bar spectra are displayed in the top and center panels of Figure 4d, respectively, facilitating optimal transport analysis. Before the OT calculation, we normalize the intensities so that their sum equals one for each spectrum, indicating the possibility of treating the spectra as discrete probability density functions.[34] The optimization of the transport plan, a Q matrix, involves minimizing the total work (W), Eq. 18, for transforming intensities from their positions within the M spectrum (top panel of Figure 4d) to form the $\tilde{C}_x^{ED}$ spectrum (central panel of Figure 4d). The different coloring of the bars within the M spectrum aids tracking the transport process for reconstructing the $\tilde{C}_x^{ED}$ spectrum.

Hence, an optimal transport plan minimizes W according to:

$$W = \sum_{i,j} c_{ij} q_{ij} \tag{18}$$

where the q_{ij} represents the Q elements, indicating the intensity amount transferred from the υ_i position to construct that at $\tilde{\omega}_j^{\tilde{C}_x}$ (bottom of Figure 4d), and c_{ij} denotes the one-dimensional distance, specifically $\left|\upsilon_i - \tilde{\omega}_j^{\tilde{C}_x}\right|$, covered by this intensity during the transport from υ_i to $\tilde{\omega}_j^{\tilde{C}_x}$. Each i-th row of Q sums up the corresponding i-th intensity in the M, while each j-th column sums up the corresponding j-th intensity in $\tilde{C}_x^{ED}$. The optimal transport plan provides the minimal W, which is the transport distance.

In the concluding step for structure identification, the computed structures are ranked based on their cross correlation and distances from the measured spectrum of interest using the above-mentioned three metrics. The cross correlation, average Euclidean, Manhattan, and minimal optimal distances, normalized by the corresponding maximum values, are illustrated using a color map.

The analysis was mostly performed using MATLAB (MathWorks, USA), utilizing the functions "xcorr", "assignmunkres", and "linprog" for cross correlation, Kuhn-Mukres, and optimal transport calculations, respectively. These functions are available in Signal Processing, Optimization, and Sensor Fusion and Tracking Toolboxes, respectively.

References

(1) Hanwell, M. D.; Curtis, D. E.; Lonie, D. C.; Vandermeersch, T.; Zurek, E.; Hutchison, G. R. Avogadro: An Advanced Semantic Chemical Editor, Visualization, and Analysis Platform. *J. Cheminform.* **2012**, *4*, No. 17.

(2) Golan, A.; Mayorkas, N.; Rosenwaks, S.; Bar, I. Raman Spectral Signatures as Conformational Probes of Gas Phase Flexible Molecules. *J. Chem. Phys*. **2009**, *131*, 024305.

(3) Shachar, A.; Mayorkas, N.; Sachs, H.; Bar, I. The Conformational Landscape of 2-(4-Fluorophenyl)-Ethylamine: Consequences of Fluorine Substitution at The *Para* Position. *Phys. Chem. Chem. Phys.* **2017**, *19*, 510−522.

(4) Mayorkas, N.; Sachs, H.; Schütz, M.; Ishiuchi, S.-I.; Fujii, M.; Dopfer, O.; Bar, I. Structural Motifs of 2-(2-Fluoro-Phenyl)-Ethylamine Conformers. *Phys. Chem. Chem. Phys*. **2016**, *18*, 1191−1201.

(5) Mayorkas, N.; Malka, I.; Bar, I. Ionization-Loss Stimulated Raman Spectroscopy for Conformational Probing of Flexible Molecules. *Phys. Chem. Chem. Phys*. **2011**, *13*, 6808−6815.

(6) Mayorkas, N.; Izbitski, S.; Bernat, A.; Bar, I. Simultaneous Ionization-Detected Stimulated Raman and Visible−Visible−Ultraviolet Hole-Burning Spectra of Two Tryptamine Conformers. *J. Phys. Chem. Lett.* **2012**, *3*, 603−607.

(7) Mayorkas, N.; Bernat, A.; Izbitski, S.; Bar, I. Vibrational and Vibronic Spectra of Tryptamine Conformers. *J. Chem. Phys.* **2013**, *138*, 124312.

(8) Mayorkas, N.; Cohen, S.; Sachs, H.; Bar, I. Photofragment Ionization-Loss Stimulated Raman Spectroscopy of a Hydrated Neurotransmitter: 2-Phenylethylamine−Water. *RSC Adv.* **2014**, *4*, 58752−58757.

(9) Rahimi, R.; Shachar, A.; Bar, I. Experimental/Computational Study on the Impact of Fluorine on the Structure and Noncovalent Interactions in the Monohydrated Cluster of *ortho*-Fluorinated 2-Phenylethylamine. *J. Am. Chem. Soc*. **2022**, *144*, 8337−8346.

(10) Shachar, A.; Mayorkas, N.; Bar, I. Structural Features of Monohydrated 2-(4-Fluorophenyl) Ethylamine: A Combined Spectroscopic and Computational Study. *Phys. Chem. Chem. Phys*. **2017**, *19*, 23999−24008.

(11) Rahimi, R.; Saban, N.; Bar, I. Synergistic Spectroscopic and Computational Characterization Evidencing the Preservation or Flipping of the Hydroxyl Group of 2-Phenylethyl Alcohol upon Single and Double Hydration. *J. Am. Chem. Soc.* **2023**, *145*, 18455−18467.

(12) Zhao Y.; Truhlar, D. G. The M06 Suite of Density Functionals for Main Group Thermochemistry, Thermochemical Kinetics, Noncovalent Interactions, Excited States, and Transition Elements: Two New Functionals and Systematic Testing of Four M06-Class Functionals and 12 Other Functionals. *Theor. Chem. Account*. **2008**, *120*, 215–241.

(13) Becke, A. D. Density-Functional Thermochemistry. III. The Role of Exact Exchange. *J. Chem. Phys.* 1993, *98*, 5648-5652.

(14) Lee, C.; Yang, W.; Parr, R. G. Development of the Colle-Salvetti Correlation-Energy Formula into a Functional of the Electron Density. *Phys. Rev. B* **1988**, *37*, 785−789**.**

(15) Chai, J. D.; Head-Gordon, M. Systematic Optimization of Long-Range Corrected Hybrid Density Functionals, *J. Chem. Phys*. 2008, *128*, 084106.

(16) Hariharan, P. C.; Pople, J. A. The Influence of Polarization Functions on Molecular Orbital Hydrogenation Energies. *Theor. Chim. Acta*, **1973**, *28*, 213–222.

(17) Clark, T.; Chandrasekhar, J.; Spitznagel, G. W.; Schleyer, P. V. R. Efficient Diffuse Function-Augmented Basis-Sets for Anion Calculations. 3. The 3-21+G Basis Set for 1st-Row Elements, Li-F, *J. Comp. Chem.* **1983**, *4*, 294−301.

(18) Kendall, R. A.; Dunning, T. H., Jr.; Harrison, R. J. Electron Affinities of the First-Row Atoms Revisited. Systematic Basis Sets and Wave Functions. *J. Chem. Phys.* **1992**, *96*, 6796−6806.

(19) Grimme, S.; Ehrlich, S.; Goerigk, L. Effect of the Damping Function in Dispersion Corrected Density Functional Theory. *J. Comput. Chem*. **2011**, *32*, 1456−1465.

(20) Hanson-Heine, M. W. D. Benchmarking DFT-D Dispersion Corrections for Anharmonic Vibrational Frequencies and Harmonic Scaling Factors. *J. Phys. Chem. A* 2019, *123*, 9800–9808.

(21) Frisch, M. J.; Trucks, G. W.; Schlegel, H. B.; Scuseria, G. E.; Robb, M. A.; Cheeseman, J. R.; Scalmani, G.; Barone, V.; Petersson, G. A.; Nakatsuji, H.; Li, X.; Caricato, M.; Marenich, A. V.; Bloino, J.; Janesko, B. G.; Gomperts, R.; Mennucci, B.; Hratchian, H. P.; Ortiz, J. V.; Izmaylov, A. F.; Sonnenberg, J. L.; Williams-Young, D.; Ding, F.; Lipparini, F.; Egidi, F.; Goings, J.; Peng, B.; Petrone, A.; Henderson, T.; Ranasinghe, D.; Zakrzewski, V. G.; Gao, J.; Rega, N.; Zheng, G.; Liang, W.; Hada, M.; Ehara, M.; Toyota, K.; Fukuda, R.; Hasegawa, J.; Ishida, M.; Nakajima, T.; Honda, Y.; Kitao, O.; Nakai, H.; Vreven, T.; Throssell, K.; Montgomery, J. A., Jr; Peralta, J. E.; Ogliaro, F.; Bearpark, M. J.; Heyd, J. J.; Brothers, E. N.; Kudin, K. N.; Staroverov, V. N.; Keith, T. A.; Kobayashi, R.; Normand, J.; Raghavachari, K.; Rendell, A. P.; Burant, J. C.; Iyengar, S. S.; Tomasi, J.; Cossi, M.; Millam, J. M.; Klene, M.; Adamo, C.; Cammi, R.; Ochterski, J. W.; Martin, R. L.; Morokuma, K.; Farkas, O.; Foresman, J. B.; Fox, D. J. *Gaussian 16*, Revision B.01; Gaussian, Inc.: Wallingford CT, 2016.

(22) Polavarapu, P.L. Ab Initio Vibrational Raman and Raman Optical Activity Spectra. *J. Phys. Chem*. **1990**, *94*, 8106–8112.

(23) Mayorkas, N.; Sachs, H.; Schütz, M.; Ishiuchi, S.-I.; Fujii, M.; Dopfer, O.; Bar, I. Structural Motifs of 2-(2-Fluoro-Phenyl)-Ethylamine Conformers. *Phys. Chem. Chem. Phys*. **2016**, *18*, 1191−1201.

(24) Shachar, A.; Mayorkas, N.; Sachs, H.; Bar, I. The Conformational Landscape of 2-(4-Fluorophenyl)-Ethylamine: Consequences of Fluorine Substitution at The *Para* Position. *Phys. Chem. Chem. Phys.* **2017**, *19*, 510−522.

(25) Shachar, A.; Mayorkas, N.; Bar, I. Structural Features of Monohydrated 2-(4-Fluorophenyl) Ethylamine: A Combined Spectroscopic and Computational Study. *Phys. Chem. Chem. Phys*. **2017**, *19*, 23999−24008.

(26) Rahimi, R.; Saban, N.; Bar, I. Conformational Mapping, Interactions, and Fluorine Impact by Combined Spectroscopic Approaches and Quantum Chemical Calculations. *J. Phys. Chem. Lett*. **2024**, *15*, 3658–3667.

(27) Smith, S. M.; Markevitch, A. N.; Romanov, D. A.; Li, X.; Levis, R. J.; Schlegel, H. B. Static and Dynamic Polarizabilities of Conjugated Molecules and their Cations. *J. Phys. Chem. A* **2004,** *108,* 11063–11072.

(28) Barone, V.; Biczysko, M.; Bloino, J. Fully Anharmonic IR and Raman Spectra of Medium-Size Molecular Systems: Accuracy and Interpretation. *Phys. Chem. Chem. Phys*. **2014**, *16*, 1759−1787.

(29) Scott, A. P.; Radom, L. Harmonic Vibrational Frequencies: An Evaluation of Hartree-Fock, Møller-Plesset, Quadratic Configuration Interaction, Density Functional Theory, and Semiempirical Scale Factors. *J. Phys. Chem*. **1996**, *100*, 16502-16513.

(30) Kashinski, D. O.; Chase, G. M.; Nelson, R. G.; Di Nallo, O. E.; Scales, A. N.; VanderLey, D. L.; Byrd, E. F. C. Harmonic Vibrational Frequencies: Approximate Global Scaling Factors for TPSS, M06, and M11 Functional Families Using Several Common Basis Sets. *J. Phys. Chem. A* **2017**, *121*, 2265−2273.

(31) Keating, M. E.; Bonnier, F.; Byrne, H. J. Spectral Cross-Correlation as a Supervised approach for the Analysis of Complex Raman Datasets: The Case of Nanoparticles in Biological Cells. *Analyst* 2012, **137**, 5792−5802.

(32) Kuhn, H. W. The Hungarian Method for the Assignment Problem. *Nav. Res. Logist. Q*. **1955**, *2*, 83–97.

(33) Munkres, J. Algorithms for the Assignment and Transportation Problems. *J. Soc. Ind. Appl. Math*. **1957**, *5*, 32–38.

(34) Seifert, N. A. Prozument, K.; Davis, M. J. Computational Optimal Transport for Molecular Spectra: The Fully Discrete Case. *J. Chem. Phys*. **2021**, *155*, 18410.

(A)

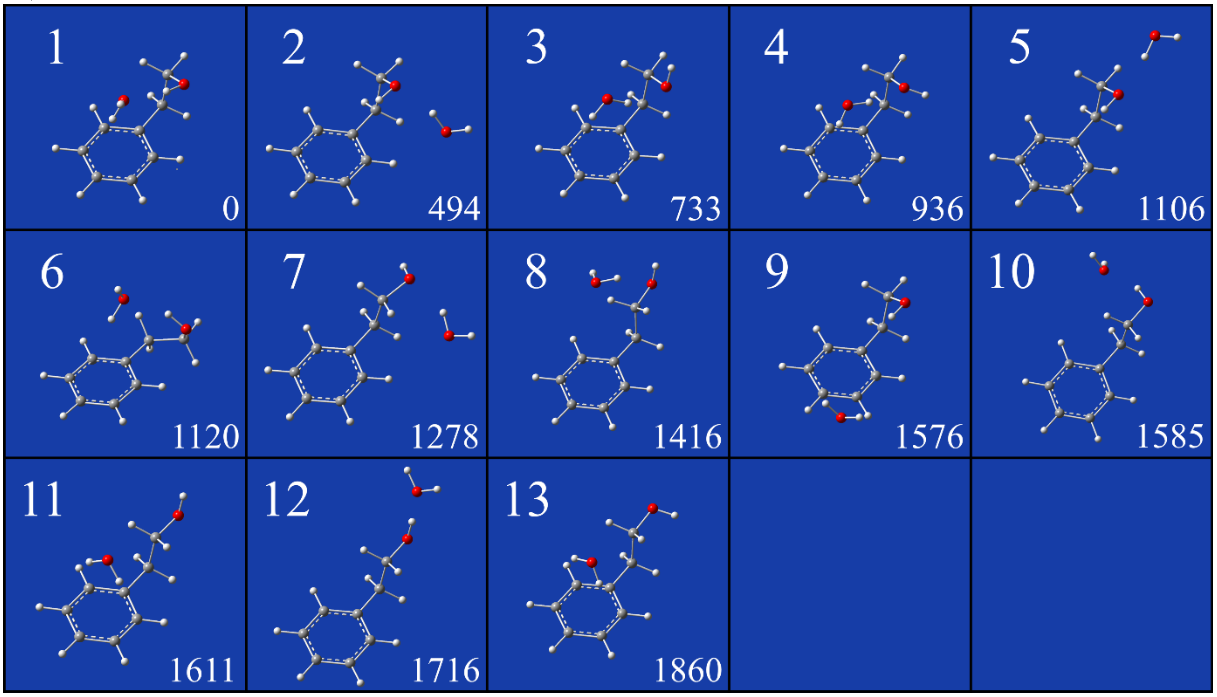
1
0
2
494
3
733
4
936
5
1106
6
1120
7
1278
8
1416
9
1576
10
1585
11
1611
12
1716
13
1860

(B)

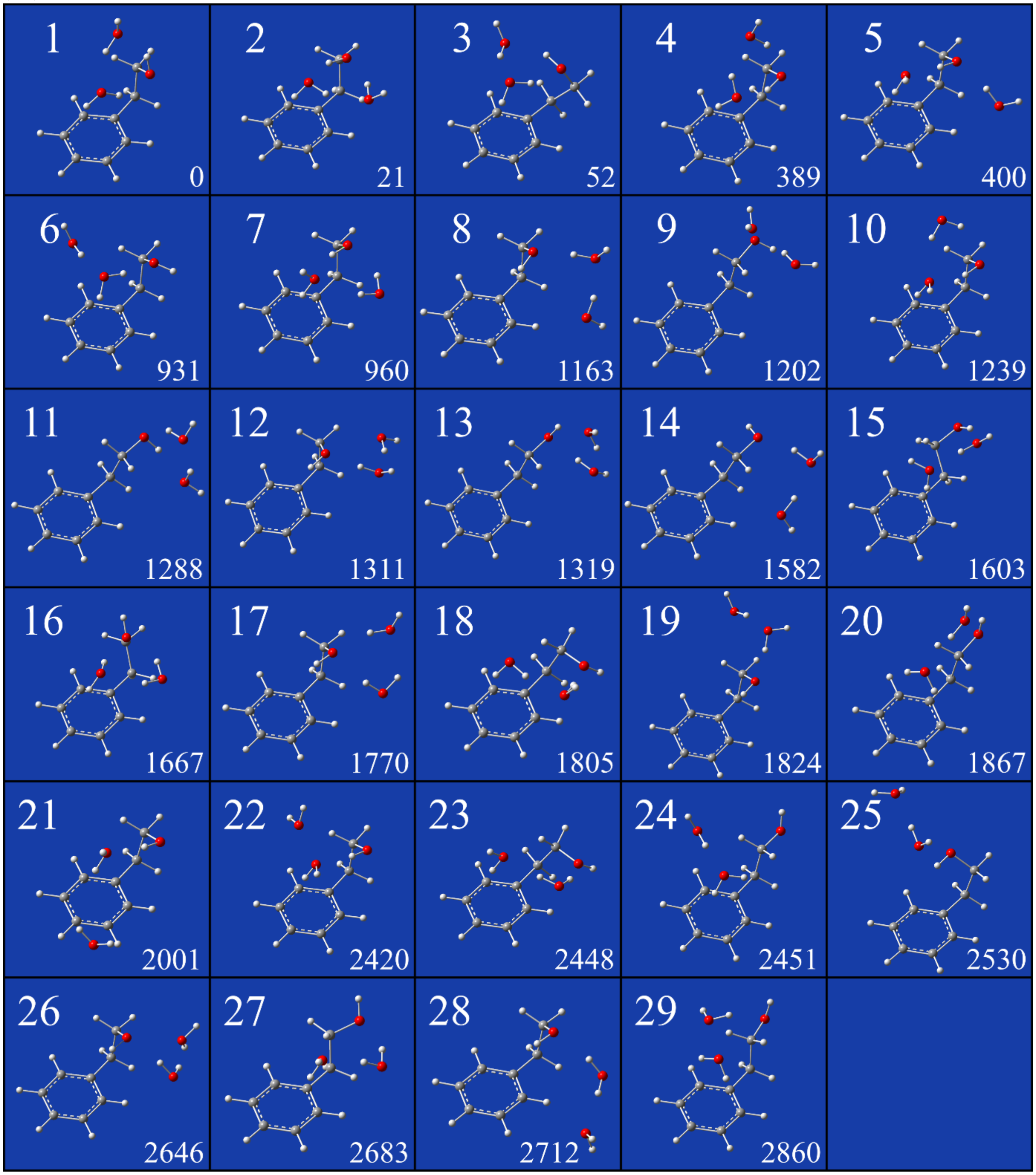
1
0
2
21
3
52
4
389
5
400
6
931
7
960
8
1163
9
1202
10
1239
11
1288
12
1311
13
1319
14
1582
15
1603
16
1667
17
1770
18
1805
19
1824
20
1867
21
2001
22
2420
23
2448
24
2451
25
2530
26
2646
27
2683
28
2712
29
2860

(C)

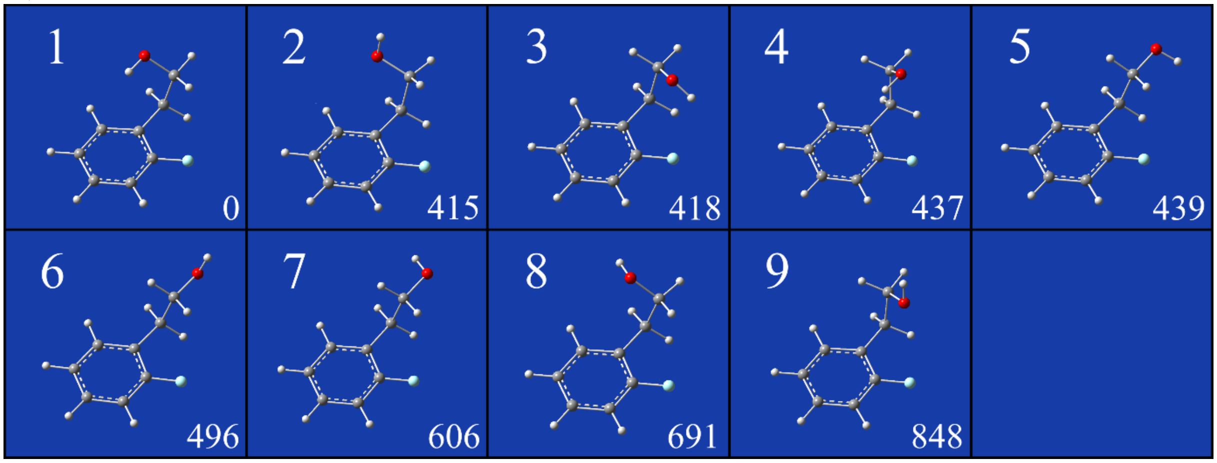

Figure S3. Geometries of the global and local minima of conformers of 2-phenylethyl alcohol (A) mono- and (B) di-hydrates and of (C**)** 2-(2-fluorophenyl)ethyl alcohol in the ground electronic state, S_0, as determined following full structural optimizations at the M06-2X-D3/6-311++G(d,p) level of theory with zero-point vibrational energy corrections. The numbers in the upper left corner label the energetic ranking and those in the lower right corner calculated relative electronic energies in cm^{-1}.

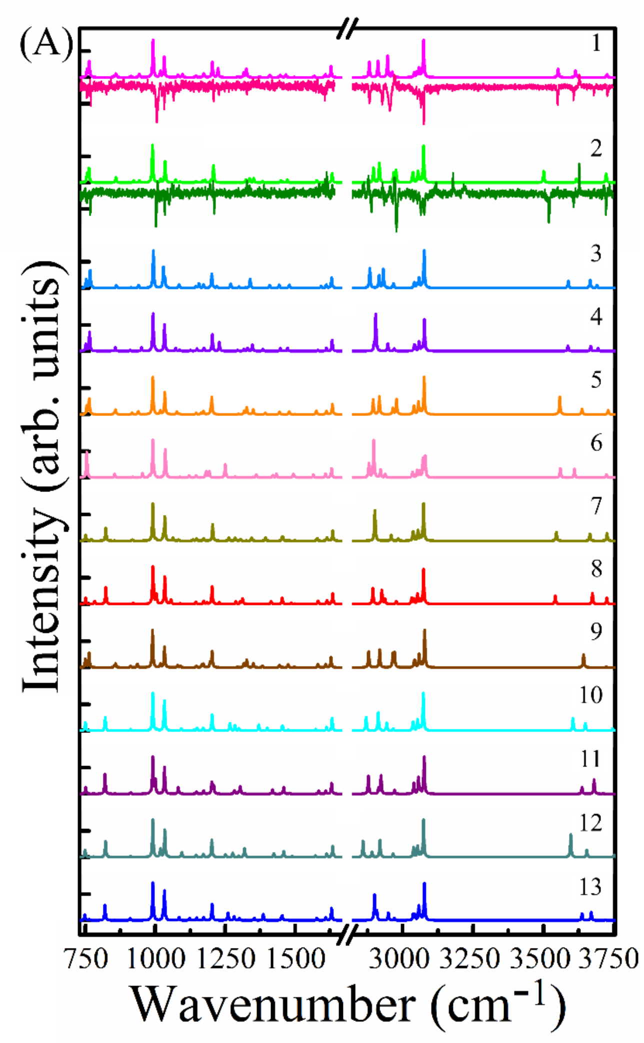
(A)
1
2
3
4
5
6
7
8
9
10
11
12
13
Intensity (arb. units)
750
1000
1250
1500
3000
3250
3500
3750
Wavenumber (cm^{-1})

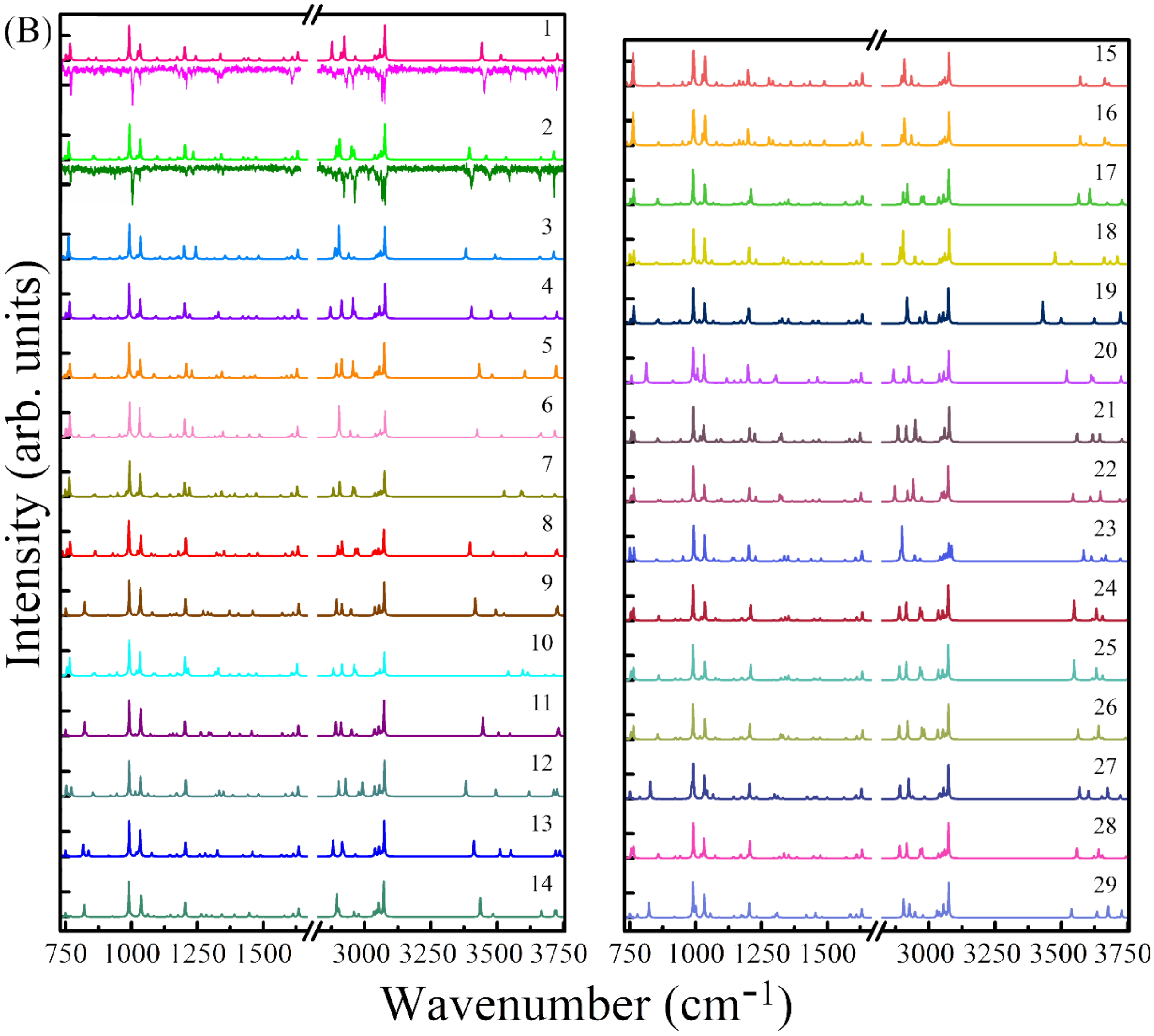
(B)
Intensity (arb. units)
Wavenumber (cm^{-1})
750 1000 1250 1500 3000 3250 3500 3750
1
2
3
4
5
6
7
8
9
10
11
12
13
14
15
16
17
18
19
20
21
22
23
24
25
26
27
28
29

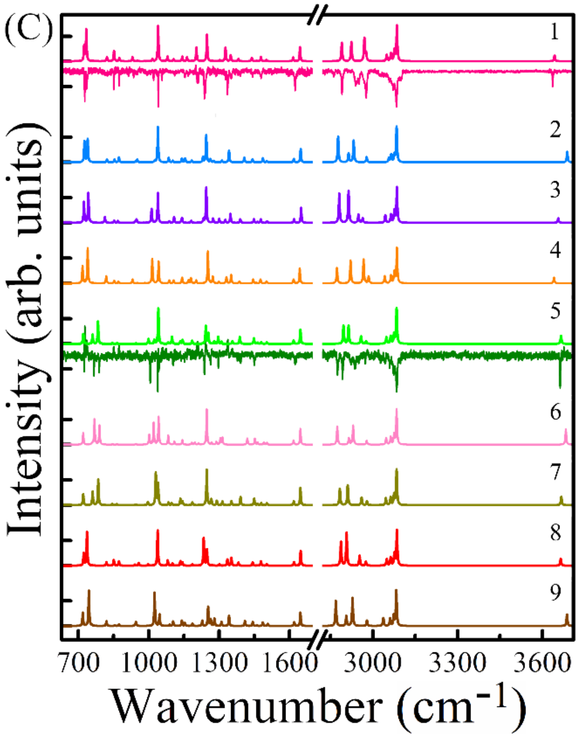

Figure S4. Measured ionization-loss stimulated Raman spectra pointing downward and scaled harmonic Raman spectra upward for conformers of 2-phenylethyl alcohol (A) mono- and (B) di-hydrates and (C**)** 2-(2-fluorophenyl)ethyl alcohol. The predicted signatures are for the corresponding optimized structures in Figure S3 (labeled by the same numbers) and calculated at the M06-2X-D3/6-311++G(d,p) level of theory and convolved with Lorentzian lines [full width at half-maximum of 4 cm^{-1}]. Panels including measured and calculated spectra show matching spectra, relying on the distance metrics in Figure 6. Measured features pointing up are gain lines of contributing global conformers of specific species.

Data 1.txt

Data 1.xlsx

Data 1. Database including the conformers and mass-to-charge ratio (*m*/*z*) used for our measurements of ionization-loss and -gain stimulated Raman features (frequencies and normalized intensities) and corresponding visually matched calculated harmonic frequencies at different levels of theory [M06-2X/6-311++G(d,p), M06-2X-D3/6-311++G(d,p), M06-2X-D3/cc-pVTZ, B3LYP/6-311++G(d,p), B3LYP-D3/6-311++G(d,p), B3LYP-D3/cc-pVTZ, ωB97X-D/6-311++G(d,p), and ωB97X-D/cc-pVTZ] computed from the resultant optimized structures, mode types or frequency ranges, and links to the source articles of the measurements

Data 2.txt

Data 2.xlsx

Data 2. Database including the conformers and mass-to-charge ratio (*m*/*z*) for measurements of ionization-loss stimulated Raman features (frequencies and normalized intensities) of 2-phenylethyl alcohol (PEAL) mono- and di-hydrates, and 2-(2-fluorophenyl)ethyl alcohol and corresponding quantitatively and visually matched Raman signatures (frequencies and normalized intensities) calculated at the M06-2X-D3/6-311++G(d,p) level of theory. The quantitative matching results from calculation of Euclidean and Manhattan distances, implementation of the Kuhn-Munkers algorithm and computation of the average Euclidean/Manhattan distances between frequencies and intensities of measured and calculated spectra
